# Advanced Nanoscale Approaches to Single-(Bio)entity Sensing and Imaging

**DOI:** 10.3390/bios8040100

**Published:** 2018-10-26

**Authors:** Marta Maria Pereira da Silva Neves, Daniel Martín-Yerga

**Affiliations:** 1 REQUIMTE/LAQV, Instituto Superior de Engenharia do Porto, Instituto Politécnico do Porto, 4200-072 Porto, Portugal; marta.neves@graq.isep.ipp.pt; 2 Department of Chemical Engineering, KTH Royal Institute of Technology, 100-44 Stockholm, Sweden

**Keywords:** single-entity, single-molecule, single-cell, nanoprobes, nanopores, nanoimpacts, nanoplasmonics, nanomachines

## Abstract

Individual (bio)chemical entities could show a very heterogeneous behaviour under the same conditions that could be relevant in many biological processes of significance in the life sciences. Conventional detection approaches are only able to detect the average response of an ensemble of entities and assume that all entities are identical. From this perspective, important information about the heterogeneities or rare (stochastic) events happening in individual entities would remain unseen. Some nanoscale tools present interesting physicochemical properties that enable the possibility to detect systems at the single-entity level, acquiring richer information than conventional methods. In this review, we introduce the foundations and the latest advances of several nanoscale approaches to sensing and imaging individual (bio)entities using nanoprobes, nanopores, nanoimpacts, nanoplasmonics and nanomachines. Several (bio)entities such as cells, proteins, nucleic acids, vesicles and viruses are specifically considered. These nanoscale approaches provide a wide and complete toolbox for the study of many biological systems at the single-entity level.

## 1. Introduction

Conventional analytical methods usually provide average information about the properties of an ensemble of entities, which is a perfectly valid vision for many studies. These techniques assume that all entities are identical, but many systems present heterogeneities or stochastic events that could govern the average response. In these methods, the properties of individual entities are masked, averaged out or invisible, missing important information that could be relevant in many (bio)chemical processes. Single-entity detection approaches [1,2,3,4] would allow for the study of properties of individual entities providing richer information, capable of revealing heterogeneities and analysis of stochastic events within different systems. There are different types of single-entities that can be considered from a chemical point of view, the dimensional scale being the most important factor. The atom would be the ultimate chemical entity, with molecules and particles of larger dimensional scale. From a biochemical point of view, biomolecules such as proteins and nucleic acids and other entities such as vesicles, viruses or cells can be considered. Combining single-entity detection approaches with biochemical matter in order to achieve single-(bio)entity sensing and imaging could make a tremendous impact: Moreover, it would open up different new horizons in the study of fundamental biomolecular processes, diagnostic capabilities of diseases at an early stage or even a deeper understanding of many pathogenesis processes, among many other possible applications. Heterogeneity is prevalent in biological systems ranging from molecules [5] to cells [6], tissues, organs and organisms [7]. For instance, heterogeneous behaviour plays an important role among cancerous cells within the same tumour [8] or different molecular structures could be observed for the same protein as a result of translation or transcription errors [5]. However, this information is neglected with conventional average measurements and may have important implications in different processes. The significance of this field can also be readily understood since the detection of single entities is the ultimate sensitivity limit (for that specific entity). In general, single-entity methods provide a unique way to see and address different biological issues of interest for the life sciences.

It is worth mentioning that the detection of different types of entities will entail different techniques and approaches. Detection of single atoms (with sizes in the order of pm) is only possible with the most advanced state-of-the-art techniques [9], and as a general rule, the dimensions of the single entity to be detected may be inversely proportional to the intricacy of the employed techniques. Optical methods are the most common for single-entity studies and have a long history and significance in the advancement of the field. Although, in general they do not use nanoscale tools to enhance the detection/imaging and, therefore, they are out of the scope of this review, it is worth to briefly mention their general possibilities. Numerous reviews have discussed the foundations and applications of optical methods for single-entity detection and imaging [5,10,11,12,13,14] and the interested reader is referred to them. Optical microscopy using incoherent white light has been widely employed to obtain morphological information from individual cells since their dimensions are usually at the microscale [15]. To get richer or subcellular information, a more sensitive technique such as fluorescence microscopy is needed, which has allowed for studying and revealing numerous cellular processes. Fluorescence microscopy also enables the detection of single molecules [10] under specific conditions such as diluted samples to guarantee that only one molecule is in resonance in the volume probed by the laser source, and providing a high signal-to-noise ratio (limiting background signal and using highly-sensitive fluorophores and instrumental systems). An interesting approach involves time-resolved measurements, which allows single-entity tracking to study trajectories and motion [14]. Unfortunately, diffraction effects limit the spatial resolution of the optical techniques (around 200 nm with state-of-the-art instruments) and impede single-molecule detection in complex samples with closely spaced emitters [16]. The diffraction limit has been overcome with the development of super-resolution optical microscopy [13,17], awarded with the Nobel Prize in Chemistry in 2014 [16]. A range of optical techniques are included in this definition, but in general, they allow for detecting single emitters at different spots in consecutive time periods, building a reconstructed image of the whole sample with nanoscale resolution. These techniques have been invaluable to study numerous biological mechanisms at the single-entity level.

In this review, we focus on several promising nanoscale approaches for sensing or imaging of single-(bio)entities: nanoprobes, nanopores, nanoimpacts, nanoplasmonics and nanomachines. Although these are quite heterogeneous topics, they cover a good range of the most advanced approaches for studies at the single-entity level. Nanotechnology can help in different ways to enable the possibility of single-entity detection with enhanced characteristics or in setups not possible with macroscale tools. Nanoscale objects could interact with individual entities since they may have dimensions close or even below to those of the entities, and in some cases the interaction could actually be one entity by one nanoscale object. They can also provide enhanced properties to increase the sensitivity, decreasing the number of entities that can be detected and enabling single-entity detection. Functionalisation of nanoscale objects with specific receptors or materials can also boost the possibilities to detect entities with higher performance. The impressive progression of nanotechnology research in the last few years has allowed for addressing numerous (bio)chemical issues from a nanoscale perspective, and the sensing and imaging of single-(bio)entities has also benefited from nanoscale advances. Some nanoscale approaches to single-(bio)entity detection such as Atomic Force Microscopy (AFM) or nanowire transistors have been omitted in this review since they have been widely and recently covered elsewhere. However, these approaches will be briefly introduced to give the reader a bigger picture. AFM [18] is a very established technique for imaging single entities. The sensing mechanism lies in a physical interaction between the AFM tip and the sample surface (typically by force attraction or repulsion), and the tip is scanned along the sample to obtain an image of the surface. The low dimensions of the tip and the precision of the micro-positioning system result in a high spatial resolution, even achieving atomic resolution (Ångstrom level), making this technique ideal for single-entity probing. Since the sensing mechanism is typically based on mechanical forces, the technique can be used for imaging conducting and non-conducting samples. AFM tip apex has nanoscale dimensions and it provides great spatial resolution. Functionalisation with different materials such as CO [19], graphene [20] or specific biomolecules [21,22] can enhance the imaging possibilities of the technique and provide chemical selectivity. The high sensitivity and spatial resolution of the AFM enables the study of single entities [23,24]. Many applications have been reported on this topic and several reviews have widely covered biochemical systems [25,26,27,28,29] from single molecules to living cells, therefore, AFM will not be covered in this review. Nanowire Field-Effect Transistors (FETs) are transistors where a nanowire is acting as the gate electrode forming an electric channel between the drain and source electrodes [30]. Conductance of these devices is controlled by changes in the charge at the channel, and therefore, it enables the detection of charged (bio)chemical species bound to the nanowire, which would change the conductance [31]. The use of nanowires increases the surface-to-volume ratio and the change of electronic conductance may be sensitive enough to enable single-molecule detection. Several materials such as In_2_O_3_ [32], silicon or carbon nanotubes [33] have been used for the nanowires. Modification of the nanowire with adequate biological receptors provides a good selectivity to the technique. Point-functionalised carbon nanotubes can serve as highly sensitive detection since a discrete point is created where the molecular attachment is performed, which enables detection of individual binding and unbinding events of single molecules [34,35]. Several studies at the single-entity level have been reported [36,37,38]. However, several recent reviews have been published where nanowire sensors are widely discussed [30,39,40].

This comprehensive review focuses on several nanoscale approaches allowing detection or imaging of single-(bio)entities. These techniques provide a wide range of possibilities to study fundamental biological processes, mechanisms or even quantification at the single-entity level. Some of these approaches are more established than others, but they are currently being developed and are reaching an excellent state that will enable to address numerous heterogeneous applications. For these reasons, a review combining these different techniques but with the common goal of single-entity detection may be valuable to the scientific community. In each section, we briefly introduce the most important characteristics for the different nanoscale approaches to provide the reader with their foundations and operating principles. Then, we describe the application of the nanoscale approaches to advanced studies of single-(bio)entities with special emphasis on recent developments. Single-entities considered in this review mainly include cells, proteins, nucleic acids, viruses or vesicles since they provide a wide range of the most important biological entities. Several reviews covering aspects of these nanoscale approaches for specific or general applications have been published over the years. They are cited in the respective sections and the reader is recommended to follow them to gain a deeper understanding of the general techniques and characteristics. In this review, we concentrate in describing the possibilities for single-(bio)entity sensing and imaging. Finally, we conclude with the most interesting remarks and challenges of the nanoscale approaches towards single-entity detection and we try to describe some of the possible directions that these topics could take in a near future.

## 2. Nanoprobes

The term probe is usually referred to a sharp object, with a specific functionality on the tip, which can be employed for probing (sensing) a sample. Nanoprobes have dimensions at the nanoscale enabling in situ localised measurements with high spatial resolution and at low confined sample volumes since the devices can be positioned in specific places of the sample. The small dimensions of the nanoprobe tip makes them highly compatible with biological living systems since they are minimally invasive and enable the possibility to study sub-entity processes such as organelles in individual cells. These characteristics are ideal for biosensing/imaging of single-entities applications, especially for single-cell analysis, although single-nanoparticle or single-molecule sensing using nanoprobes have also been reported.

Nanoprobes can be used in two main configurations: (a) discrete positioning of the probe within the sample, and (b) in scanning mode (scanning probe microscopy techniques). In the first case, the nanoprobe is placed in a specific location in close proximity to the sample with a positioning device and usually with the aid of an optical microscope. After placing the probe, the measurement is performed at that specific site (discrete measurements). In the scanning mode, the nanoprobe is moved over the sample surface (typically in xyz directions) and controlled measurements are automatically performed at different positions within the same sample. This mode leads to more versatile measurements since it allows the sensing and imaging of the sample, with the possibility to obtain information on chemical composition and morphology (depending on the employed technique and the type of nanoprobe) and enable the detection of heterogeneities on the surface. The scanning of the nanoprobe can be performed by different modes: continuous or in approach-hold-withdraw (hopping) [41] mode. In the latter, a measurement can be performed at different locations across the surface, acquiring spatially-resolved data from a series of x-y pixels. This mode is very useful for rough surfaces since it decreases the chances of probe breaking by colliding with surface features, which could happen more easily in continuous mode.

**Figure 1:**
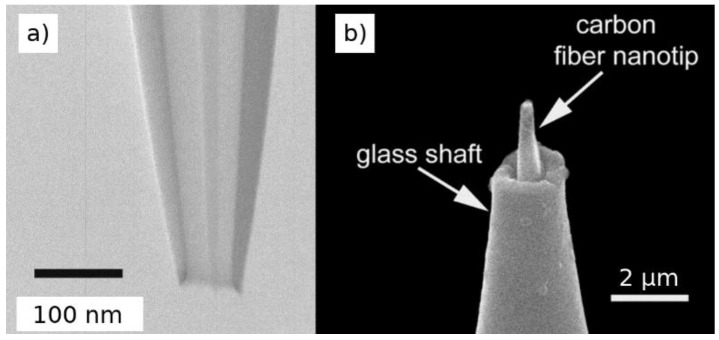
Electron microscopy images of (**a**) a quartz nanopipette and (**b**) a nanoelectrode fabricated with a carbon fiber and pulling of a glass pipette. Adapted with permission from [46,47] Copyright 2016 American Chemical Society and 2014 John Wiley and Sons.

Two main representative nanoprobes (Figure 1) that can be used for single-(bio)entity detection are covered in this review: (a) needle-type nanoelectrodes (typically fabricated with a glass capillary filled with a conducting material) and (b) nanopipettes, where the capillary is kept empty and filled with electrolyte. They are certainly versatile and have been applied to study a vast number of biological processes with notable studies at the single-cell level, but with the possibility to achieve spatial resolution at the single nanoparticle [42] or biomolecule level [43]. Spatial resolution is mainly due to the size of the probe tip (including the active part and the covering), and thus it is envisioned that improved fabrication techniques could lead to nanoprobes with enhanced spatial resolution extending the possibility to probe smaller single (bio)entities. Tip size is also another factor to consider for achieving less invasive and destructive measurements, and nanoprobes have demonstrated a good performance in live cells studies without significant perturbation. Another limiting aspect is the instrumental sensitivity since it is difficult to differentiate ultralow electric signals coming from small individual entities from background noise. However, the main limitation of these techniques is the low specificity. In the case of Scannine Electrochemical Microscopy (SECM), the signal typically comes from a redox reaction, which could be due to different chemical species, although enhanced selectivity can be achieved by modification of the SECM tip with adequate receptors or specific materials [44]. Time of analysis is another concern since acquiring high-quality images could take a significant amount of time, increasing the chances of sample evolution during the measurement. Several strategies have been reported to achieve high-speed imaging to address this issue [45].

### 2.1. Fabrication and Functionalisation of Nanoprobes

Nanoprobes can be fabricated with different materials and physical properties (e.g., size, shape). One of the most established methods for nanoprobe fabrication is pulling glass capillaries with a laser puller to reduce down the size of one of the capillary apertures to the nanoscale. This method is quite versatile since it can be applied for fabrication of nanoelectrodes if a metallic microwire is placed inside the capillary before the pulling process [48] or for nanopipettes, if the capillary is left empty. The size of the nanoprobe tip can be controlled with high precision by controlling the pulling conditions, and dual or theta-barrel capillaries can also be used for fabrication of multifunctional nanoprobes. Carbon nanoelectrode can be in situ generated by pyrolysis inside the capillary [49]. Other fabrication methods have been described elsewhere [50,51].

**Figure 2:**
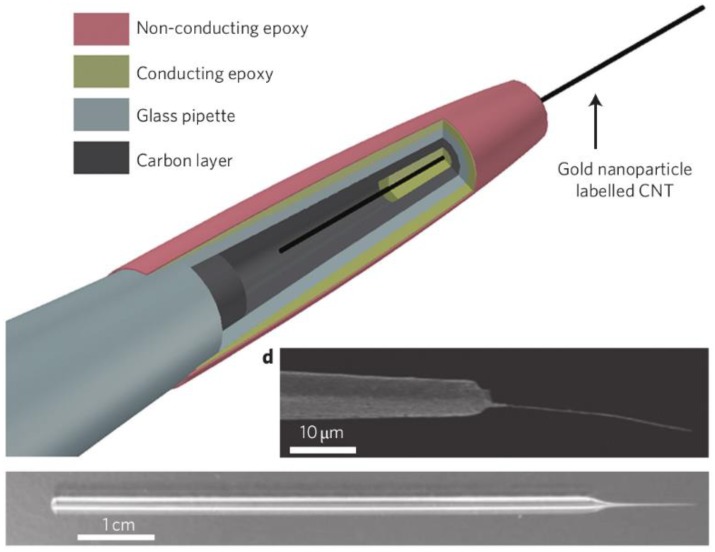
Nanoelectrode fabricated with a single carbon nanotube introduced in a glass pipette coated with conducting materials. Adapted with permission from [54] Copyright 2010 Nature Springer.

Modification of nanoprobes with specific materials can provide new functionalities and open up new applications. Although specific examples will be cited in sections below, it is interesting to describe some initial approaches to demonstrate how the functionalisation can improve the sensing capabilities of nanoprobes. For instance, nanoelectrodes fabricated by filling a nanopipette aperture with pyrolytic carbon have been modified to enhance its functionalities. Modification with Prussian blue has been reported in order to enhance the detection capabilities towards H_2_O_2_ [52]. In this case, the nanoelectrode was etched by electrochemical cycling in NaOH in order to make space in the glass tip, and then the Prussian blue was electrodeposited into the etched nanocavity. Electrodeposition of Pt has also been reported on carbon disk-shaped nanoelectrodes to monitor oxygen reduction reaction since the Pt is able to catalyse this reaction [53]. A single carbon nanotube could be attached into a carbon-coated micropipette (Figure 2) to create a nanoscale electrode using the carbon nanotube achieving spatial resolution of 100 nm for minimally invasive probing of cells [54]. Micro/nanoelectrodes have also been modified with ion-selective layers to provide enhanced detection of ions by potentiometry [55,56].

Nanopipettes can also be modified to provide new functionalities. Several works have been reported describing functional nanopipettes [50] or modification with proteins/enzymes [57,58]. For instance, hollow pipettes can be coated with metallic thin films in order to prepare a combined nanoelectrode/nanopipette device consisting of a metallic ring electrode but still leaving the nanoscale aperture of the capillary in the middle. These systems can be used for delivering solutions at the picoliter range near or inside individual cells [59] or to perform combined nanoelectrode/nanopipette techniques such as scanning ion conductance microscopy (SICM) or scanning electrochemical microscopy (SECM) [60]. A lipid monolayer can also be formed at the tip opening of a dual-channel pipette. The device is held into a lipid solution (in organic solvent) while applying a small potential between two quasi-reference counter electrodes (QRCEs) placed in each channel in order to attach the lipid monolayer. Then, the modified devices can be used in an aqueous solution with another lipid monolayer on the surface to form and perform studies in/with a lipid bilayer [61].

Multifunctional nanoprobes can be prepared/modified with distinct combined functionalisations in order to boost the amount of information that is obtained during the measurement. For instance, the deposition of gold nanoparticles on nanoelectrodes formed by a single carbon nanotube has provided the possibility of performing localised surface-enhanced Raman scattering (SERS) analysis using the nanoprobe [54]. Individual barrels of multichannel capillaries as used in nanopipettes can be specifically functionalised. One barrel of dual-barrel capillaries was filled with carbon to form a nanoelectrode and the other barrel was left empty and filled with electrolyte and a quasireference counter electrode [62,63]. This allows to have in the same nanoprobe a sensing electrode (for electrochemical measurements) and a nanopipette (for ionic conduction measurements/manipulation). A similar procedure can be performed on four-barrel capillaries, forming two carbon electrodes and two electrolyte-filled channels on diagonally opposite sides of the capillary [64,65]. In this configuration, the two electrodes can be used to drive local electrochemical reactions while an ion current can flow between the hollow electrolyte-filled barrels to control and position the probe on the sample with high precision.

### 2.2. Nanoprobes Employed in Static Mode

When working in static mode (non-scanning mode), the nanoprobe is positioned close to the sample to be probed and a measurement is performed at a specific discrete position. The operating principle when the nanoprobe is a nanoelectrode is that it acts as the working electrode in an electrochemical cell, also integrated by a reference electrode. The electrochemical response (several techniques are possible) could be employed to get information about the concentration of analyte in the sample. The current measured is usually very small due to the nanoscale dimensions of the electrode leading to a minimal ohmic drop, which enables the use of a two-electrode system in contrast to conventional setups. The small dimensions also lead to low background currents associated with the double layer charging and to an increased rate of the mass transport. The latter allows to measure kinetic information of fast electron transfer processes. The operating principle for nanopipettes is the following: a QRCE is placed inside the nanopipette filled with electrolyte and another QRCE is placed outside in the solution that contains the sample. A bias potential is applied between both electrodes, which generates an ionic current flowing through the nanopipette aperture. When the nanopipette is close to the sample, a change in the magnitude of the ionic current is produced due to the surface properties of the sample, which thus can be sensed. Nanoelectrodes or nanopipettes in static mode have been used in numerous (bio)sensing applications at the single-cell level. Most popular applications are the intracellular detection of reactive oxygen and nitrogen species (ROS/RNS), metabolites or to study processes such as exocytosis.

#### 2.2.1. Detection of Reactive Oxygen and Nitrogen Species in Single Cells

Reactive oxygen species (ROS) and reactive nitrogen species (RNS) regulate cellular functions and have an essential role as signalling molecules [66]. The cellular levels of these species are an important marker of oxidative stress, which may be the origin of substantial cellular damage even turning into carcinogenesis processes [67]. Needle-type nanoelectrodes in combination with electrochemical monitoring are excellent tools to detect ROS/RNS in single cells [68]. These species can be effectively detected using Pt-modified electrodes since this metal is able to catalyse the electrochemical reactions, achieving a high sensitivity. In this sense, several works detecting ROS/RNS inside single cells with nanoelectrodes have been reported. For instance, Pt black was electrodeposited inside an etched nanocavity of a Pt nanoelectrode and used for detection ROS/RNS species [69]. The capacity of this nanoelectrode was tested in vitro using solutions of H_2_O_2_, peroxynitrite anion (ONOO^−^), nitric oxide (NO) and nitrite anion (NO_2_^−^) as model systems. The platinisation (Pt nanodeposits) was essential in order to obtain a sensitive response to ROS/RNS, demonstrating good electrocatalytic properties. Then, the device was inserted inside a murine macrophage and the intensity of oxidation bursts was monitored. Etched carbon nanoelectrodes modified with electrodeposited Pt black were also employed for ROS/RNS quantification in individual human breast cells [70]. In this case, different cell models were employed: healthy human breast epithelial cells and two metastatic breast cancer cell lines. A higher amount of ROS/RNS in the most aggressive phenotype cells and insignificant amounts in healthy cells were observed, suggesting a strong correlation between intracellular generation of ROS/RNS and cell malignancy. The same nanoprobe was also employed to study carcinogenesis in cells after stimulation with a specific agent, observing the onset of an inflammatory process. Nanowire electrode have also been employed for ROS/RNS sensing in single cells [71]. These nanoelectrodes were fabricated using a SiC nanowire, which confers a very high mechanical stability and then coated with conductive carbon in a core-shell structure. Pt electrodeposition was also performed in order to obtain electrodes able to detect the oxidative species in an effective way. Then, the nanowire electrode was inserted into phagolysosomes of single living macrophages. When the cells were stimulated, the generation of ROS/RNS was detected as current spikes by amperometric detection.

#### 2.2.2. Detection of Exocytosis in Single Cells

Secretion of (bio)chemical messengers from an emitting cell into the extracellular space is called exocytosis [68]. These messenger species are carried out by secretory vesicles, and when they fuse with the cell membrane, some of its content is released. This process is essential to cell function and can provide important information about cell states. In case of exocytosis of neurotransmitters such as catecholamines from synaptic vesicles, those analytes can be detected by electrochemical methods, and therefore, nanoelectrodes are suitable to study the exocytosis in living cells. For instance, a technique that was called intracellular vesicle electrochemical cytometry has been reported [72]. A nanoelectrode is inserted into the cell and the collision of individual vesicles with the electrode leads to the lysis of the vesicle and the release of the neurotransmitters, which are electrochemically detected. This technique has been used to measure individual vesicles in single PC12 cells using a carbon fibre nanoelectrode. Experiments with the nanoelectrode placed on top of the cell (externally) to detect exocytosis events were also performed, observing that only a fraction of the neurotransmitters are released during exocytosis [72]. A similar experiment was performed using nanoelectrodes formed by Au nanodisks of 6 nm diameter. Dopamine release was monitored from single living vesicles in single PC12 cells with high spatial resolution, allowing subcellular resolution for evaluating the exocytosis rate [73]. Amperometric detection of neurotransmitter release have also been reported for single synapses. A conical carbon nanofiber electrode was employed to monitor individual vesicular exocytosis events in real time and their kinetics from inside single synapses with high spatio-temporal resolution [47] as well as to record postsynaptic potential in combination with glass nanopipette electrodes [74].

#### 2.2.3. Detection of Other Analytes in Single Cells

Nanoprobes in static mode can be used to detect other interesting chemical species involved in cellular processes that can be very relevant for many applications in the life sciences. For instance, nanoelectrodes were used to measure only a few HeLa cells confined in a single droplet in microscopic wells. The cells were identified and quantified by their alkaline phosphatase activity which leads to the formation of an electroactive product electrochemically detected at the nanoelectrode [62]. Electrochemical detection of oxygen inside cells can be very useful for monitoring oxygen consumption or the redox-state of the cell. Carbon disk-shaped nanoelectrodes functionalised with Pt were employed for monitor oxygen consumption outside and inside a brain slice. The use of nanoscale electrodes minimises the perturbation to the tissue because oxygen depletion is reduced. This approach was successfully applied to individual neurons both in tissue and isolated [53]. In a different approach, a polished Pt nanoelectrode was used to penetrate human breast cells for intracellular voltammetry of oxygen reduction (to probe redox properties of the cell) [75]. No apparent damage was observed in the cell during the measurements due to the small dimensions of the probe. The last two approaches were also employed in a SECM configuration in order to achieve the electrochemical imaging of cell surfaces.

Nanopipettes functionalised covalently with glucose oxidase were employed for localised detection of glucose in single cells [58]. The inner walls of the nanopipette was modified with poly-lysine, activated with glutaraldehyde to attach glucose oxidase. In presence of glucose, the enzymatic reaction produces a local pH drop, which changes the impedance at the nanopipette tip. This system was employed to detect glucose in human fibroblasts and metastatic breast cancer cell lines, observing higher intracellular glucose levels in individual cancer cells. A *lab-on-a-tip* system, which authors named as nanokit, was also employed for intracellular detection of glucose in single living cells [76]. A capillary sputtered with a Pt thin film on the external walls, forming a ring electrode was employed as nanoprobe. The nanoprobe was filled with electrolyte and the reagents needed to perform a specific reaction. In case of glucose detection, the electrolyte contained glucose oxidase (GOx). The nanoprobe can be placed inside a cell and femtoliter amounts of the solution can be released into the cell. Glucose would react with the GOx and would form H_2_O_2_, which can be electrochemically detected by the nanoelectrode. This smart system was also employed to detect sphingomyelinase activity in cells when the nanoprobe was filled with a solution of sphingomyelin, alkaline phosphatase, and choline oxidase.

A multifunctional nanoprobe formed by attaching a single carbon nanotube to the tip of a glass micropipette was employed to interrogate cells down to the single organelle level [54]. The nanotube can be filled with magnetic nanoparticles for remote movement to transport nanoparticles and attoliter fluids to and from precise locations. The nanoprobe can be used for electrochemical measurements, and when modified with gold nanoparticles for SERS detection. This device was employed to test changes in mitochondrial membrane potential at the single-organelle level.

### 2.3. Scanning Nanoprobe Techniques

In scanning probe techniques, the nanoprobe is moved along the sample to obtain spatially resolved images. These techniques provide some interesting features such as the possibility to image heterogeneities of individual entities and ensembles at the single-entity level to study interactions between individual entities. Depending on the technique and configuration, multifunctional information such as the sample topography, quantification of analytes or surface charge can be obtained. In this review we will introduce two scanning techniques using nanoprobes: scanning electrochemical microscopy (SECM) and scanning ion conductance microscopy (SICM). They are certainly versatile and have been applied to study a vast number of biological processes with notable studies at the single-cell level.

#### 2.3.1. Scanning Electrochemical Microscopy

Scanning Electrochemical Microscopy (SECM) [77,78] is a scanning probe technique that uses an ultrasmall needle-like electrode as a mobile probe to obtain localised information of a substrate in a solution. Substrates can be conducting, semiconducting or insulating materials, perturbing the electrochemical response in different ways. This technique provides information about the substrate as topography and heterogeneities across the surface, in contrast to macroscale electrochemical methods where the response is the average from the whole substrate. Different electrochemical techniques can be used to measure the properties of the substrate and, therefore, quantification of analytes may be possible exploiting the concentration dependence with the measured current. SECM has been extensively used with ultramicroelectrodes (dimensions typically around 1–25 µm) from Pt, Au or C materials and extensive literature has been reported. These dimensions are enough for a variety of applications, for example to probe many individual cells, but the use of nanoscale probes can significantly boost the spatial resolution to get information about smaller entities. The use of nanoscale electrodes has also other advantages such as the increase of the mass transport to the electrode, very low ohmic drops and capability to measure electrochemical reactions at individual nanoobjects such as nanoparticles [79].

SECM measurements can be performed in different ways considering the approach to detect the surface. Initially, simple constant-height and constant-current modes were used. In constant-height mode, the probe is kept at a specific height from the sample plane during the imaging process. Since the sample topography can be heterogeneous, the real tip-sample distance can change, which together with variation of the sample activity lead to changes in the current at the tip. This configuration has several issues, especially using nanoscale probes since the probe needs to be particularly close to the sample (tip radius and tip-sample distance are related), and it can become difficult with heterogeneous samples. In constant-current mode, which avoids this issue, the positioning system automatically moves the nanoprobe in the z-direction to keep the electrode current constant, allowing for obtaining topographical information. However, in both methods, the measured signal is dependent on the tip-sample distance and the electrochemical activity of the sample, making difficult the discrimination of both factors. For this reason, numerous strategies to decouple the distance-to-the-sample and the electrochemical information have been developed to achieve the positioning of the tip at a true constant distance to the sample since the tip closely follows the contours of the surface in a non-destructive way. These distance-control methods help to control the position of the nanoprobe with higher precision near the sample, enhancing the quality of topographical information, and allows to obtain distinctive electrochemical information without the effect of the tip-sample distance (more precise quantitative information). These methods have enabled the high-resolution imaging of complex heterogeneous samples with nanoprobes such as cellular or other biological systems. Some of these methods include shear-force [80], alternating-current [81], voltage-switching [82], standing approach [83] or the use of combined techniques such as SECM/SICM [60].

The sample can be interrogated using different strategies being the most employed the feedback, tip generation-substrate collection (TG/SC), substrate generation-tip collection (SG/TC), shielding or competitive and potentiometric modes [84]. In feedback mode, an electrochemical reaction is monitored by measuring the tip current. The signal changes when the tip is far from the sample (bulk solution is measured) and when the tip approaches the sample. Two effects can be detected: (a) if the substrate blocks the diffusion of reactant to the tip, a decreased current is measured (negative feedback), (b) if the substrate causes the regeneration of the reactant, an increased flux to the tip occurs (positive feedback). In TG/SC mode, the tip causes an electrochemical reaction and the product of this reaction suffer another reaction in the substrate. This mode is very convenient for studies of homogeneous chemical reactions such as the oxygen reduction and evolution reactions, very significant in electrocatalysis. In SG/TC mode, the substrate generates a product that is measured at the probe tip. In shielding or competitive, both the tip and substrate compete for the same reactant, and therefore, the tip can detect indirectly the activity of the substrate. For potentiometric SECM, the use of a selective electrode (ion-selective or similar) as the working probe allows to detect selective changes in potential due to the presence of the analyte at different concentration.

SECM has found applications in many fields such as imaging of surfaces (topography), surface modification or nano/micro patterning, studies of chemical catalysis, electrocatalysis, reaction kinetics, energy materials or biological systems among many others. Several reviews have been published with detailed information on SECM applications [59,84,85,86]. The excellent properties of SECM with nanoscale electrodes increases the possibilities to study biological systems such as cells or other entities (inside and outside the entity), measure sub-entity heterogeneities, understand processes between ensembles of entities or even tissues. It has also been proven powerful to study the local activity of biosurfaces (modified with proteins or nucleic acids) for sensing, gaining valuable knowledge of localised activity to understand the biochemical processes happening in biosensing devices.

#### 2.3.2. Single-Entity Detection with SECM

The most reported applications of SECM in the detection of single-(bio)entities is the sensing/imaging of single cells, with the possibility to obtain information in real time of the cell topography and particular cellular functions [86,87,88]. Imaging of the cell surface can be performed enabling the possibility to detect heterogeneities at different positions of the individual entity. Some of the general applications include quantification of molecules entering or leaving the cell, probing intracellular reactions or studying ion transport in the cell membrane [59], which allow for gaining a deep knowledge about cellular mechanisms that can have a great significance in many applications in the life sciences.

SECM was initially developed when fabrication of nanoscale probes was not implemented, and the first advances were achieved using microscale SECM probes (ultramicroelectrodes with diameters typically between 1 and 25 μm). SECM with microscale probes has been widely employed and even now that nanotechnological techniques are quite widespread, these probes are still useful in many applications. Therefore, although this review is mainly focused on nanoscale approaches to single-entity detection, some approaches to sensing/imaging of single cells with microscale SECM probes with interesting functionalisations are initially described since they have promising applications in the biosensing field and many methodologies could also be implemented with nanoscale probes. For instance, SECM microelectrodes have been modified with enzymes for highly localized measurements achieving a high specificity in analyte recognition. Functionalisation of Pt ultramicroelectrode with glucose or lactate oxidases by electropolymerisation or casting was performed in order to sense and imaging glucose or lactate in single living cells, which play an important role in cell metabolism [89]. A comparative study of enzyme immobilisation techniques such as cross-linking, electropolymerisation and physical adsorption was performed in Pt SECM ultramicroelectrodes for glucose and lactate oxidases. Th electropolymerisation method was chosen as the more convenient since it leads to higher spatial resolution, and it could be a good option also for functionalisation of nanoscale electrodes. Then, the probe was employed for SECM imaging of glucose uptake in single living cells. A voltage-switch method was used to avoid topographical contribution to the signal in order to enable quantitative measurements [44].

Very different information from single cells can be obtained from SECM analysis. For instance, the imaging of an epidermal growth factor receptor, a key membrane protein associated with cancer, was possible in a single living mammalian cell [90]. The receptor was modified with an antibody labelled with alkaline phosphatase, and a product of its enzymatic reaction was detected with the SECM electrode to localise the specific membrane proteins and estimate the density of these proteins in the cell surface. Intracellular redox activity of individual living cells can be a marker of cancer development and SECM has proven useful to measure the redox balance of different cells, observing significant differences between healthy human breast epithelial cells and highly metastastic cancer cells [91,92]. SECM is also interesting for studies of cancer treatment and it has been used for the assessment of multidrug resistance-related protein in patterned adenocarcinoma cervical cancer cells. This may help evaluate the effectiveness of treatment administration strategies [93].

The advantages of using nanoscale probes for sensing/imaging were already mentioned, with special emphasis for the enhanced spatial resolution. In this case, the distance between the nanoelectrode and the sample must be closer than using microscale probes and usually is in the order of the tip radius. Several studies of single cells have been reported using SECM with nanoelectrodes. Perhaps, the first report of nanoscale imaging of biological entities was described by Fan and Bard [43]. A nanoscale probe from a scanning tunnelling microscope was employed as nanoelectrode. With this setup, it was possible to imaging DNA and proteins at the single-entity level (resolution down to 1 nm) on a mica substrate. Nanoscale SECM probes fabricated by pulling a Pt wire inside a capillary were employed to image individual living human breast epithelial cells with lateral resolution down to 100 nm, enabling the imaging of the cell membrane with high resolution. The cell membrane potential was estimated by measuring voltammograms inside and outside the cell, which was used to evaluate the effect of cell exposition to several agents [75]. A voltage-switching mode was employed to enable the simultaneous acquisition of high-quality topographical and electrochemical images of living cells. The system was useful to visualise the topography of different kind of cells and membrane proteins on A431 cells. Detection of neurotransmitters released from single PC12 cells in real time as current spikes in an amperometric measurement was also possible, although using microscale electrodes to increase the collection efficiency [82]. Pt nanoelectrodes have been used to detect oxygen-related functions in single cells with SECM. For instance, the oxygen consumption of single HEK293 cells was successfully studied by an interesting approach. Potential pulses were employed in order to minimise the extent of the reaction at the nanoelectrode to enable the detection of very low oxygen consumption by individual cells [94]. SECM nanoprobes have also been demonstrated for simultaneous imaging and manipulation of single cells. The imaging of individual normal rat kidney cells was performed with SECM achieving better results than using confocal laser scanning. A cathodic potential was applied to the nanoelectrode in order to increase the local concentration of hydroxide ions near the cell, which led to the cell necrosis, subsequently imaged by SECM with the same probe [95]. Multifunctional SECM nanoprobes have been developed in order to obtain simultaneous information from the same sample. An optical fibre electrode combining a nanoelectrode (35 nm radius) and an optical probe (aperture less than 170 nm) was fabricated with the possibility of simultaneous electrochemical, topographic and optical imaging. The system was employed for multifunctional mapping of single cells under physiological conditions [83]. In a similar system, this time using a shear-force mode as probe positioning feedback was used for simultaneous topographical, fluorescence and electrochemical imaging of gene-transfected single HeLa cells. The system was able to measure the cellular expression activity of secreted alkaline phosphatase and green fluorescence protein outside and inside the cell, respectively [80].

#### 2.3.3. Scanning Ion Conductance Microscopy

Scanning Ion Conductance Microscopy (SICM) [96] is a scanning-probe technique that uses a nanopipette filled with an electrolyte, where a quasireference counter electrode (QRCE) is also placed. Another QRCE is placed on the electrolyte solution outside of the nanopipette and a bias potential (small potential difference) is applied between the two QRCEs. The potential difference induces the flow of an ionic current between the two QRCEs through the nanopipette tip. When the nanopipette approaches the surface, the resistance at the nanopipette tip changes resulting in a different ionic conduction depending on the distance and surface properties of the sample. The precise positioning of nanopipettes allows high-resolution imaging (localised measurements) of the sample surface and combined with a distance-control feedback method, generation of three-dimensional topographical images is possible. Distance-control methods have the same significance than in SECM, allowing a better control of the tip-sample distance and enabling the simultaneous acquisition of topographical and activity information, converting SICM in a multifunctional tool beyond topography [97]. Shear-force [98], alternating-current [99], pulse mode [100] and hopping [101] modes have been proposed to achieve this goal. SICM has been great for measuring/imaging surface charge of samples. The ion current flowing by the pipette between the two QRCE electrodes is dependent on the polarity of the applied bias and can suffer surface-induced rectification depending on the surface properties, which can be used to create functional images of surface charge in the sample [102]. Although getting direct chemical information from SICM measurements is not easy, many (electro)chemical reactions consume or produce ions, modifying the local conductivity, that can be detected by the SICM probe, and thus, the technique can be used to image ion fluxes coming from reactions at a surface, with special relevance for electrochemical reactions [103]. A smart self-referencing hopping mode protocol as positional feedback system for simultaneous visualisation of topography and electrochemical fluxes at and around single nanoparticles was also used [104]. This smart mode lies in using the tip current to detect the approach to the sample since a decrease in current is expected when the tip-sample distance decreases. When the system detects the surface, the nanopipette is retracted a fixed distance (about 20 nm) to minimise effects of the tip on the mass transport or diffusion layer of the nanoparticles, achieving the measurement of electrochemical ion fluxes coming from the nanoparticles always at the same distance. This process is repeated at different positions (“pixels”) of the sample by scanning the nanoprobe to obtain topographical and electrochemical activity information across the surface, which can open a myriad of new possibilities for SICM imaging.

SICM has been applied to study a large number of interfacial issues reaching the imaging of electrocatalytic reactions at the nanoscale and single-particle level [104]. In the biosensing/bioimaging field, SICM has been widely applied in many studies with biological samples [50,105,106]. As other nanoprobe techniques, it is very appropriate to study cellular processes in living cells at the single-entity level since it is a non-contact technique that avoids cell deformation [107] Combined with the low invasive nanoscale probes, it has also been applied to intracellular studies of living cells. Morphology is one of the most studied cell properties by SICM, but it has also proven useful to detect other processes such as release of messenger signals through the cell membrane or ion channels. Some of the most relevant studies using SICM at the single-cell level will be addressed.

##### Topographical Imaging of Single Cells

Perhaps, the most employed application of SICM imaging of single cells is the determination of cell topography, which can provide information about the cell properties and the cell response to different stimuli [106]. This has been demonstrated in numerous cases using microscale pipettes, obtaining interesting and versatile information of cell properties such as the dynamics of cell volumes [108], changes in cell membrane morphology associated with exocytosis [109] or localisation of single active ion channels in membranes of living cells [110], among many other examples. However, the utilisation of nanopipettes leads to an enhanced spatial resolution for imaging and identification of smaller topographical features. In combination with the less invasive characteristics of nanoprobes, SICM could be advantageous to AFM on very soft samples such as living cells, especially considering the long-term imaging stability [107]. Hopping mode is a good feedback method to allow a precise non-contact imaging of heterogeneous structures such as cells. This has been applied to nanoscale imaging of different kinds of cells [111] with spatial resolution down to 20 nm [101]. Imaging of proteins in single cells have also been reported. Proteins secreted from human endothelial cells during exocytosis induced by a chemical agent were imaged by SICM [112]. The technique was also employed for imaging proteins protruding from the plasma membrane of living cells at the single-protein level [113] or single virus particles on the surface of cell membranes [114].

##### Imaging Surface Charge of Individual Cells

**Figure 3:**
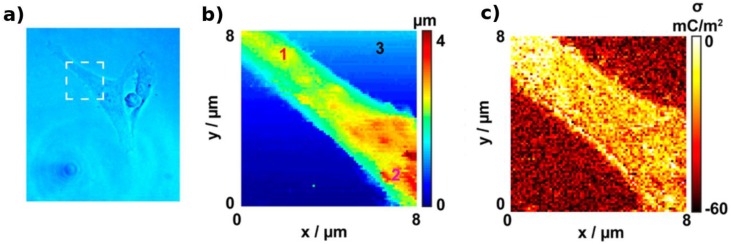
Imaging of a cell with (**a**) optical microscopy; (**b**) topographical data from scanning ion conductance microscopy (SICM) measurements and (**c**) surface charge data from SICM measurements. Adapted with permission from [116] Copyright 2016 American Chemical Society.

Simultaneous surface charge and topography of living cells have also been determined by nanoscale SICM. For instance, a bias modulated configuration was used to decouple the topographical and surface information allowing the quantitative imaging of nanoscale surface charge of *Zea mays* root hair and human adipocyte cells. This technique allowed seeing distinct local surface charge distributions across the surface of the cells due to the high spatial resolution provided by the nanoscale SICM tips and distance-control feedback method [115]. A potential-pulse chronoamperometric approach was employed with the previous system, which avoided the lock-in detection leading to an enhanced data acquisition rate. This strategy was employed for imaging of surface charge heterogeneities in neuron-like PC12 cells (Figure 3) under physiological conditions [116].

##### Study of Ionic Transport in Single Cells

Understanding ion transport in single cells is important since it can provide relevant information in biological cell processes with relevance in biomedical sciences. Potentiometric-SICM (P-SICM) was developed by Baker’s group for imaging different ionic transport properties in cells. P-SICM uses a double-barrel nanopipette with two QRCEs in each compartment. One of the electrodes monitors ion current as a distance-control feedback and the other electrode measures the local electric field respect to the reference electrode in the bulk solution. This technique has been used to study different transport processes in cells such as paracellular permeabilities in tight junctions [117] or to differentiate processes happening in bicellular and tricellular tight junctions [118]. The technique combined with a hopping-mode as feedback to position the probe close to the sample could be employed for simultaneous topography imaging and visualisation of heterogeneous ion transport in epithelial cells [119].

##### Multifunctional Imaging with SICM Combined Techniques

Multifunctional nanoprobes combining nanoelectrodes and nanopipettes have also been employed for imaging of living cells using combined SECM/SICM techniques [65]. This configuration allows to obtain simultaneous high-resolution topographical information with the nanopipette and electrochemical information by probing the sample with the nanoelectrode [120]. For instance, this multifunctional probe that was used for imaging of live cells and to probe electrochemical activity from enzymes [60]. The open barrel of the multifunctional probe can also be used as a localised delivery tool to the sample. This approach was employed to local delivery of K+ to PC12 cells to stimulate neurotransmitter release, which was detected by the nanoelectrode [63]. A similar approach was also employed for spatially resolved mapping of the uptake of molecules at living cells, observing heterogeneities in uptake rates across several regions of single *Zea mays* root hair cells with nanoscale resolution [121].

## 3. Nanopores

Nanopores are nanoscale apertures able to transduce the presence of analytes into an intelligible signal. They are usually supported in a membrane (artificial or biological) that separates two compartments filled with electrolyte, only communicating through the small opening of the membrane. In nanopore sensing, the analyte can be detected when passing through the nanopores between one compartment to the other. To achieve the translocation of the analyte, a voltage is usually applied between two non-polarisable electrodes placed in each compartment leading to the flow of an ionic current through the pore. This situation generates a strong potential gradient close to the nanopore leading to the transport of the molecule through it, which in the end is a result of an interplay between electrophoretic, electroosmotic and diffusion effects [122]. Individual translocation events through the nanopore leads to transient (stochastic) changes in the measured signal, therefore a statistical analysis is often needed and average data is used to analyse chemical properties. Frequency, duration, amplitude and even the shape of the measured signal can be used to obtain different chemical information about the molecule passing the nanopore such as the size, charge, structural conformation or concentration [123,124]. If the molecule is large enough, even the detection of molecular heterogeneities in a single molecule across its structure is possible, opening the door, for example, to DNA sequencing.

The small dimensions of a nanopore enable single-entity detection since many molecules have an appropriate size to translocate through the nanopore one-at-a-time. Advances in nanofabrication and molecular biology have allowed for developing a vast number of versatile nanopore systems (artificial, biological and hybrids) with specific sizes and functionalities able to address many sensing applications. Despite of the high potential for single-entity sensing, nanopores have yet some practical challenges that must be addressed before achieving a widespread use in many applications. For instance, electrical noise can become an issue [125], especially in solid-state nanopores, and could limit the signal-to-noise ratio and, therefore, the sensitivity [126]. This is particularly important when using high bandwidth filters, which are needed to detect fast translocation events. Electrical capacitance of the device also affects the noise level and the possibility to detect faster transient responses. Some approaches have been developed in order to reduce the capacitance and improve the detection [127,128]. Stability of the nanopore system could also be relevant in biological pores. The actual pore system or the lipid membrane usually need specific conditions for adequate operation and harsher conditions could lead to structure changes and malfunctioning. Stability of solid-state pores could also be compromised after blockage by non-specific adsorption of large biomolecules. Translocation events of small molecules are usually very fast, which makes difficult their effective detection by the instrumental system, although functional nanopores to increase the interaction time between pore-analyte and the use of high-bandwidth filters are alternatives to enable the detection of smaller molecules [129]. Another possible limitation of nanopore sensing is the low-throughput. Since the translocation events happen one at a time, it may become difficult to detect the passage of the analyte through the nanopore in a reasonable amount of time, especially at low concentrated samples. The lack of selectivity is also an issue since all molecules with size lower than the nanopore are able to pass through it, although the functionalisation of nanopores with appropriate receptors seems a good way to enable the selective identification of the target analyte. Some approaches have been developed to minimise these possible issues and to achieve single-entity detection of very different molecules, which will be highlighted in this review. Focus will be on interesting recent reports of nanopores sensing for single-entity detection after a brief introduction to nanopore types and the most employed detection techniques.

### 3.1. Types of Nanopores

There three main types of nanopore systems: (a) biological nanopores that you can usually find in biological systems formed by biomolecules (proteins, lipids, DNA), (b) artificial or solid-state nanopores that are synthesised by means of nanofabrication techniques and (c) hybrid nanopores [130] that normally combine a solid-state membrane with an aperture where a biological nanopore is placed. Comparisons between biological and solid-state nanopores have been described elsewhere [131,132]. Briefly, the main differences are the higher reproducibility of the biological pores, they generally enable lower translocation speeds (low aperture diameter) [131] but also a lower chemical and mechanical stability. Solid-state nanopores are more versatile for modification to increase functionalities, are compatible with different types of detection, and their integration in sensing devices can be straightforward. They also can be fabricated by atomically thin materials providing excellent spatial resolution.

#### 3.1.1. Biological Nanopores

Biological nanopores are mainly formed by proteins or protein assemblies including ion channels, porins or bacterial toxins among other systems, presenting a nanoscale aperture (pore) supported on lipid bilayers. These proteins are usually found on cell membranes and play the role of regulating the molecular transport between the cellular compartments. They have pore sizes at a scale similar to many biomolecules and have been useful for sensing of ions, small molecules, DNA or even proteins. The translocation events are usually detected by resistive pulse method (vide infra) by measuring the ionic current blockage when the analyte is passing the pore. Fabrication of biological nanopores is possible by employing standard molecular biology techniques [133]. Even though the biological nanopores are systems with specific molecular structures as found in biosystems, they can be engineered by several methodologies [123,134,135] such as site-directed mutagenesis or incorporation of specific adaptors tuning the nanopore properties for particular applications. Biological nanopores have several properties such as an excellent biological selectivity since the pore is able to sense specific targets. The reproducibility is also excellent (with atomic precision) since they present a well-controlled geometry determined by the molecular structure. The low mechanical and chemical stability are usually the biggest drawbacks since they only work under specific physiological conditions or the handling is difficult. Therefore, it is difficult to adapt these systems to manufactured devices.

The first nanopore sensing application was reported in 1994 using a biological nanopore: the α-hemolysin (α-HL) nanopore embedded in a lipid bilayer [136]. This nanopore has been widely employed in numerous sensing applications [137] and has been considered as a good model as biological nanopore. Since the first report of biological nanopores, many other systems have been demonstrated as good candidates for nanopore sensing. To cite a few: OmpG [138], ClyA [139], FhuA [140], CsG [141] or Aerolysin [142,143] nanopores. These nanopores have their specific characteristics and further information can be found elsewhere [144]. Recent and interesting sensing applications of biological nanopores will be highlighted in the next sections.

#### 3.1.2. Solid-State Nanopores

Solid-state nanopores are synthetic devices with a nanoscale aperture in a solid-state membrane. Advanced in nanofabrication techniques have enable the precise control of the nanopore size of the generation of special architectures. Therefore, they can be fabricated with tunable properties such as size or geometry from different materials, providing them with special functionalities. In fact, fabrication of nanopores with conducting metallic structures can enable new translocation detection techniques such as electron tunnelling using metallic nanogap electrodes inside the pore (vide infra). Solid-state nanopores usually resist more severe conditions (temperature, pH, ionic strength) than biological nanopores since they can have increased chemical, thermal and mechanical stability. This versatility facilitates the functionalisation of the nanopore surface to boost their characteristics, allow their integration intro manufactured devices and introduce new applications. One of the major drawbacks of solid-state nanopores is the lack of specificity since all the entities smaller than the nanopore size could translocate through it. However, surface functionalisation can be employed as a strategy to improve the selectivity by means of chemical interactions with the right molecules.

Fabrication of solid-state nanopores can be performed via different techniques, being perhaps the most predominantly used the direct milling with focused electron or ion beam [145,146] techniques. Other fabrication methods include dielectric breakdown [147], TEM drilling [148] and optical heating [149]. An interesting approach is to use DNA origami since it allows for the fabrication of 3D shapes with precise control over geometry and surface functionality [150,151]. Several general reviews have previously described fabrication techniques of solid-state nanopores [152,153,154,155] and therefore, they will not be discussed here. Different materials have been demonstrated for solid-state nanopores applications such as glass, polymers, silicon species [148] or carbon nanotubes [156], among many others. These materials present specific properties that could be more appropriate for particular applications or under different experimental conditions. Two-dimensional materials such as graphene, MoS_2_ or BN are also promising materials for the construction of nanopores since they provide ultrahigh spatial resolution [157,158,159,160,161]. For instance, these materials have a thickness comparable to the spacing between DNA nucleobases, being very appropriate for high resolution sequencing [162,163]. Glass nanopipettes have also been widely used for nanopore fabrication [164,165]. They are easily fabricated by laser pulling of a glass capillary leading to the decrease of one of the capillary apertures to nanoscale dimensions. Then, the nanopipette can be filled with electrolyte, one electrode placed in the inner compartment and the other electrode placed in an external solution. These nanopipettes can also be positioned and scanned with high precision, resulting in the SICM technique (which is described in the nanoprobes section of this review).

Functionalisation of solid-state nanopores can provide special features to create smarter devices enabling the detection of single-entities in more effective ways. A wide variety of nanopore surface modifications have been reported in the literature [153]. For instance, organosilane chemistry can be employed for modification of SiNx nanopores or thiol-based chains can be bound to gold surfaces in order to attach functional groups to the nanopore structure. This would allow for introducing groups able to modulate surface charge, respond to local conditions or provide steric effects to enhance the molecular selectivity of the nanopore. Different strategies have been employed to make nanopores responsive to local pH changes by modification with an adlayer of poly(4-vinylpyridine) [166] or by introducing different surface functionalities to the interior and exterior of the nanopore [167]. This modification allows to make pH-dependent transport through the nanopore and thus turn between on and off states depending on the local pH. Nanopores switchable by temperature changes have also been reported after modification with thermoresponsive poly(*N*-isopropylacrylamide) brushes [168].

Functionalisation of solid-state nanopores with biochemical or biomimetic receptors is a good approach to provide selective binding of a specific biomolecule in the nanopore [153]. This binding can help to control the translocation speed to improve the spatial resolution during the detection. This is interesting to identify molecular heterogeneities in a single molecule such as the possible determination of different nucleobases in a DNA molecule or to increase the translocation residence time of small molecules enabling its detection by the sensing system. Some versatile examples include the modification with a nitriloacetic acid receptor to allow the detection of stochastic binding and unbinding processes of Histidine-tagged proteins in real time [169], the attachment of DNA probe molecules for specific binding and detection of complementary DNA target sequences [170,171] or the use of aptamers to immobilise and detect single proteins [172].

Plasmonic nanopores are a special type of functionalised nanopores with metallic nanostructures placed very close or inside the aperture. These metallic nanostructures can provide enhanced optical properties or related features due to the focusing of the optical field and generation of surface plasmons. The plasmon-enhanced optical field allows to provide local heating at the nanopore even at the attoliter level [173]. This local heating can be exploited to control the rate of translocation events by thermophoresis (thermal gradient) [174,175] or to switch on or off the transport through the pore upon optical excitation [176]. The generated local heat from the plasmonic enhancement has also been exploited for in situ fabrication of nanopores with excellent size control [149,177]. Plasmonic nanopores can also enable the enhanced optical detection by SERS in combination with the control of the translocation event [178].

### 3.2. Detection of Translocation Events

**Figure 4:**
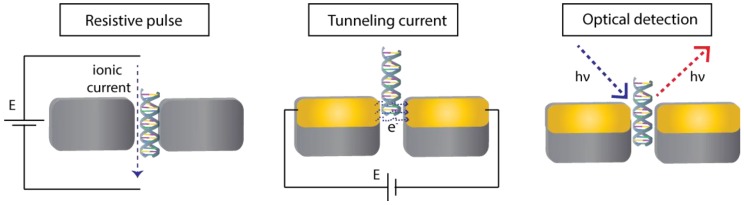
Schematics of different methods to detect the translocation events through a nanopore: resistive pulse, tunneling current and optical detection.

Translocation events need to be detected in order to get information about the chemical systems in a sample. The detection can be performed in function of time (with real-time monitoring) by exploiting different physicochemical properties (Figure 4). When a molecule goes through the nanopore, a stochastic signal is measured, whose properties can reflect the properties of the molecule passing by the nanopore. The most employed detection method is perhaps the resistive pulse method, which is based in the Coulter counter technique. This method is readily available and compatible with all nanopores systems since it uses the response coming from the same electrodes that generate the electrophoretic field to induce the migration of molecules. Detection of tunnelling currents between nanogap electrodes inside the pore is another detection approach. Optical detection by fluorescence [179], Raman spectroscopy [180] or force-based methods have also been reported [153]. In this review, the main characteristics of the resistive pulse, tunnelling current and optical detection methods are briefly described since they are the most reported in the literature.

#### 3.2.1. Resistive Pulse Detection

The molecule translocation through a nanopore is performed by applying a bias potential between two electrodes in each compartment of the system. This process generates a flow of ionic current between both electrodes and ions will pass through the pore. This ionic current is measured continuously in function of time. When the molecule translocates through the nanopore, a blockage of the ionic current usually occurs since less ions will be able to pass by the pore in that moment. This event results in a stochastic change in the measured ionic current. The frequency of the stochastic events is usually proportional to the concentration of analyte in the sample. Other parameters of the signal such as the magnitude, duration or shape can also provide more information on the molecule translocating the nanopore, such as size, composition or interactions with other molecules. Some effects that should be considered during the of electric signals are the ion current rectification [181] or the possibility to see a biphasic waveshape response under specific conditions such as high potential bias or low ionic strength solutions [182].

#### 3.2.2. Tunneling Current Detection

For electron tunnelling detection [183,184], a nanopore device is fabricated in combination with two electrodes placed at the nanopore with a small nanogap between them. A potential bias is also applied between these nanoelectrodes, and a tunnelling current can flow between both electrodes through the nanogap. When a molecule translocates through the nanopore, the electron tunnelling will change due to the perturbation. Electron tunnelling is very sensitive to the environment between the electrodes and it can provide interesting information about the electronic structure of the molecule. For instance, the tunnelling current decays with distance leading to enhanced spatial resolution and provides information about molecular size. The increased spatial resolution can provide a more efficient detection of biopolymer composition (very appropriate for sequencing applications). Since this signal is not related to the electrodes used to induce the translocation, it opens the possibility of parallel detection with nanopore arrays.

#### 3.2.3. Optical Detection

Although not as used as the electrical detection techniques, optical detection in nanopore sensing has also been reported. Localised surface plasmons generated in metallic nanomaterials after light excitation could be exploited to enhance the detection of scattered light [185]. When the molecule translocates through the pore, it will cause a change in the refractive index inside the pore that is representative of the molecule properties and will lead to a change in the detected scattered light. These stochastic optical fluctuations can be measured by dark-field microscopy with single-molecule sensitivity and have shown as good results as the resistive-pulse method, opening the possibility to have simultaneous optical and electrical detection methods. Surface-enhanced Raman scattering effect has also been exploited for nanopore detection with the possibility to use the identification potential of the Raman technique [186]. Another optical technique with single-molecule sensitivity is fluorescence, which was also reported for nanopore sensing. In this case, labelling with a fluorophore is usually needed to get a strong fluorescent signal, and a Total Internal Reflection Fluorescence (TIRF) instrument is normally employed to decrease background signals and enhance sensitivity [187,188]. In some cases, a metallic layer has also been used in the nanopore membrane to produce plasmon-enhanced fluorescence [153].

### 3.3. Single-Entity Detection with Nanopores

The properties and versatility of nanopore systems place them in the group of frontier techniques to address studies at the single-entity level. They have been widely employed for the detection of single-(bio)entities such as viruses [189,190,191] and especially for proteins and nucleic acids analysis. The high spatial resolution of the nanopore technique allows for the possibility to obtain sub-molecular information enabling the development of nucleic acid sequencing systems. In contrast, detection of single cells is not usually possible since the nanopore dimensions are lower than the typical size of cells and the cell cannot translocate.

#### 3.3.1. Nucleic Acid Detection

**Figure 5:**
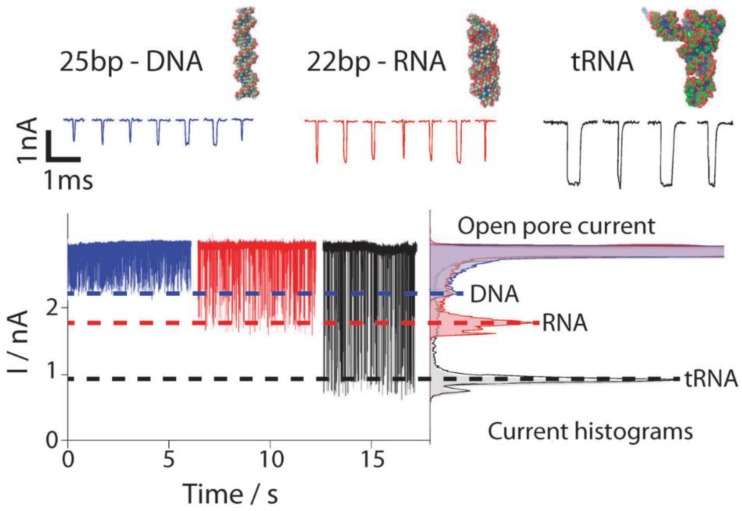
Stochastic current responses of translocation events of different DNA and RNA molecules. Shape and amplitude of the current responses were different and enabled the discrimination of the different molecules Adapted with permission from [200] Copyright 2010 Springer Nature.

Nucleic acid detection is the star application in nanopore sensing (in combination with sequencing), and numerous nanopore systems have been developed such as biological pores [192,193], solid-state nanopores with resistive-pulse detection [194], tunnelling detection [183,195], simultaneous ionic and tunnelling detection [184] and optical detection [186]. Single molecules of single-strand DNA (ssDNA) [196], double-strand DNA (dsDNA) [197] and RNA [198] have been detected with nanopores systems. The nanopore system demonstrate a great potential to even be able to detect knots in DNA structures [199] or to differentiate between DNA and RNA with different structures (Figure 5) [200].

One of the biggest issues in nanopore sensing is the control of the translocation events. On one hand, the translocation event should be slow enough leading to increased residence time of the molecule inside the nanopore to enable a more effective detection (enhanced signal-to-noise ratio). One the other hand, nanopore sensing can detect single-molecules but the probability that one specific molecule pass through the pore in a short time depends on the concentration in the solution, being very difficult to detect translocation events in samples at low concentrations. In recent years, significant and novel advances to address these issues have been developed. For instance, a double-nanopore system in which a single DNA molecule could be mechanically trapped increasing the residence time was reported [201]. Another double-nanopore system (fabricated in a double-barrel nanopipette) was used to slow down translocation rate. This was possible by the formation of a zeptoliter nanoscale droplet at the nanopipette tip, which acts as a nanobridge between the two pores and where the molecules are confined. This system increases the signal-to-noise ratio, resolution, sensitivity and limit of detection and can be universally applied to single-molecule detection of dsDNA, ssDNA, RNA and proteins [202]. A system also formed for a double-barrel nanopipette (double-nanopore) was employed in two modes of operation: (a) pore-to-pore transfer and (b) DNA molecules bridging between the two nanopores, which allowed for controlling the DNA transport and the molecular sensing with enhanced temporal resolution [203]. Kinked silica nanopore arrays were suspended over a silicon nitride aperture and used for DNA detection [204]. The kinked nanopores showed up to five-fold reduction in translocation speed and the small pore size allowed for selectively detecting ssDNA over dsDNA. Nanoscale preconfinement of translocating molecules using an ultrathin nanoporous silicon nitride membrane separated from a nanopore by a nanoscale cavity has been another approach to slow-down and reduce the variability of the translocation speed between different DNA molecules [205]. In order to enhance the low frequency translocation events with samples at low concentrations, a nanopore system fabricated with a metallised nanopipette that was able to concentrate molecules at the nanopore by dielectrophoretic trapping was developed, increasing the detection efficiency by 1000-fold and achieving detection of single-molecules at concentrations as low as 5 fM at a rate of 315 events per minute [206]. Although biological nanopores are less versatile in terms of modification possibilities, some strategies can also be used to control translocation events. It was found that the aerolysin nanopore have different sensing regions and the translocation times of oligonucleotides through the nanopore can be modulated by targeting a specific site [142].

Another recent trend in nanopore sensing is the use of plasmonic nanopores and these systems have also been applied to nucleic acid detection. The optical excitation of the plasmonic nanopores can be used for controlling the local temperature and the translocation process [207]. In situ opening of nanopores in membranes by localised heating is another application of plasmonic nanopores, which can also be used for enabling high-sensitivity optical detection of single molecules [186,208] or for multifunctional detection by simultaneous recording optical and electric signals [208], which can increases the information obtained for the same molecule.

#### 3.3.2. DNA Sequencing

**Figure 6:**
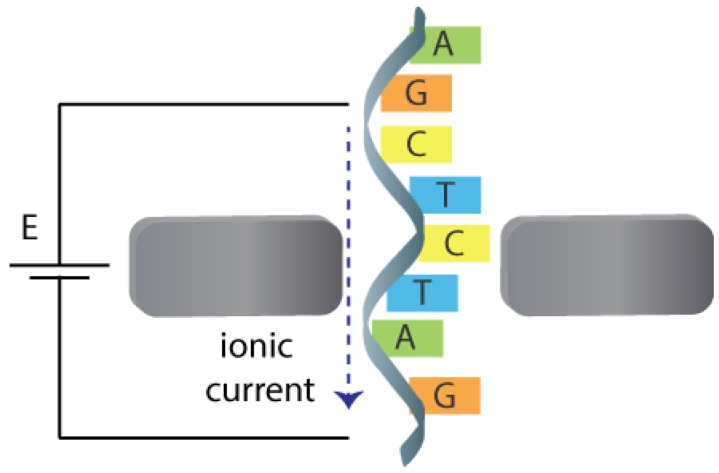
Schematics showing the spatially-resolved translocation of a DNA molecule through a nanopore for sequencing applications.

Since nanopores are able to detect nucleic acids at the single-molecule level and to identify individual nucleobases from the same molecule, DNA/RNA sequencing has been one of the most popular applications (Figure 6). Improvement of the current sequencing technology with a high-throughput, low-cost, label and amplification-free technology with real-time monitoring would be a great breakthrough for genomic studies. Nanopore sensing is a very interesting approach in order to achieve a technology with these ideal characteristics [163]. DNA sequencing using nanopores was pioneered in 1996 using an α-haemolysin biological nanopore where single-strand DNA molecules translocated in elongated conformation, and each nucleobase interacted in a different way with the nanopore leading to identifiable signals [133]. Since that remarkable advance, numerous developments have been reported for nucleic acid sequencing using nanopores [132,209,210,211,212].

**Figure 7:**
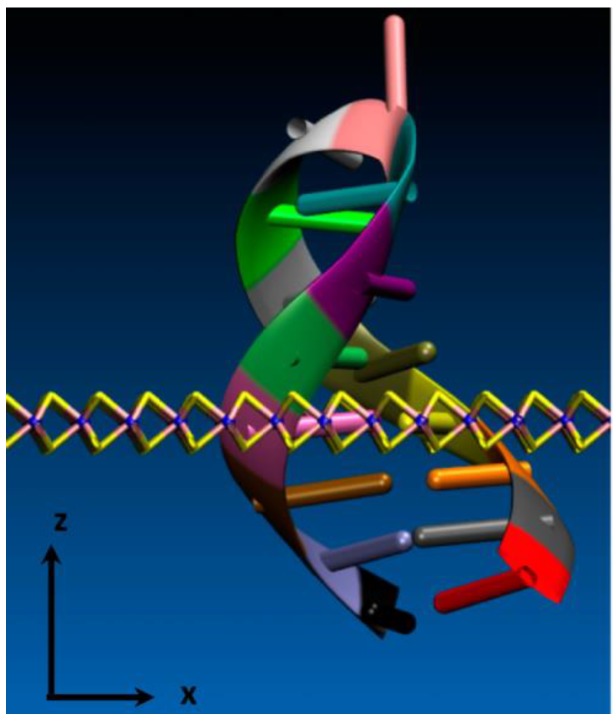
Schematic showing the dimensional scale of an atomically thin MoS_2_ nanopore respect to the size of individual nucleotides from a double strand DNA molecule. Adapted with permission from [162] Copyright 2014 American Chemical Society.

In recent years, the advances in nanopore technology have also demonstrated interesting features for nucleic acid sequencing application. For instance, the use of atomically thin 2D materials such as graphene or MoS_2_ for the membrane shows promising results since their thickness is close to the size of individual nucleobases (Figure 7), being the material with ultimate spatial resolution. Graphene materials have been employed for DNA sequencing [159], but the fast translocation speeds of DNA molecules limit their usability. An approach to address this issue was to use a viscosity gradient based on ionic liquids to control the translocation speed through MoS_2_ nanopores, achieving single nucleotide translocation speeds for DNA sequencing [213].

Plasmonic nanopores have also been employed to control the translocation speed of DNA molecules and enhance the resolution in order to achieve DNA sequencing. Interestingly, this system was able to control the translocation of the DNA molecule by discrete steps, sequentially exposing the different nucleobases to the plasmonic nanopore and allowing for high resolution detection by SERS, which showed a distinctive signal for each nucleobase [178].

Commercial nanopore technology has also been proven useful for genome sequencing in real world applications such as during the Ebola epidemic in 2015 [214].

#### 3.3.3. Protein Detection

Numerous examples of protein detection in nanopores at the single-molecule level have been reported in the literature with solid-state [215,216,217] or biological [137,138,218] nanopores. Besides the detection of the molecule itself, other properties have been evaluated such as the charge or size [219], conformational heterogeneity in protein ensembles [220] or identification of IgG antibody subclasses [169].

As previously mentioned, the small size of many proteins makes the efficient detection of the translocation events difficult, since these molecules pass through the nanopore at a very fast speed with short residence times. Advances in instrumentation technology such as the use of high-bandwidth filters can help to detect fast translocation events and enable the detection of smaller molecules [129,221]. Another trend in recent years is the use of nucleic acid carriers that can bind specific proteins to generate a biomolecular complex of higher size, slowing down the translocation time and enabling the detection through the nanopores. For instance, DNA aptamer-modified gold nanoparticles as molecular carrier to specifically bind lysozyme were described [222]. This system is thus very selective to the target protein, and the single translocation events of the complex carrier are detected in the nanopore. Double-strand DNA was also employed as molecular carrier for single-molecule protein detection. [223]. The DNA was functionalised with a specific aptamer molecule to bind proteins in human serum. Specificity is conferred by the aptamer sequence and thus by changing this sequence is possible to recognise different molecules even simultaneously (multiplexed detection) due to the unique ion current signatures of the protein-bound carriers. Other similar systems generated by DNA-based constructs were applied to detection of different proteins [224,225,226,227,228].

Approaches to slow down the translocation speed have been successfully reported in nanopore sensing of proteins at the single-molecule level. For instance, the functionalisation of quartz nanopores with specific antibodies was performed to monitor the interaction between single α-foetal proteins and its antibody at single-molecule level, after the affinity binding of both molecules in the nanopore [229]. A controllable gate voltage in a nanopore extended field-effect transistor allowed the slowing down of the transport of molecules through the nanopore. Functionalisation of the nanopore with specific receptors increased the selectivity towards the target molecules, enabling the detection of antibodies and DNA molecules [230].

Recent reports have also used biological nanopores for single protein detection. For instance, the detection of trypsin (a biomarker for pancreatitis) was achieved by monitoring the enzymatic reaction between a substrate and trypsin and detecting individual translocation events of the reaction product in an α-haemolysin nanopore. This system provides good sensitivity and selectivity by using the catalytic enzymatic reaction [231]. It was reported that an OmpG nanopore can be used to detect biotin-binding proteins after functionalisation of the rim of the nanopore with biotin ligands. The system was demonstrated for detection of avidin proteins and antibiotin antibodies [232].

## 4. Nanoimpacts

**Figure 8:**
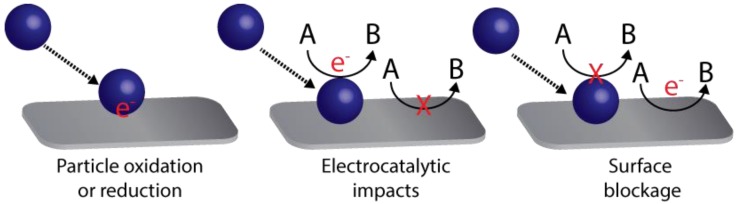
Different electrochemical approaches to detect collisions of nanoparticles.

The nanoimpact method is an emergent approach for electrochemical characterisation and stochastic analysis of single-entities [2,233]. This approach entails the electrochemical detection of individual collisions of particles against an electrode [4,234]. Although the use of macroelectrodes to detect nanoimpact events has been reported [235,236], nano/microelectrodes could be more appropriate since they can be used for the study of fast electron transfers (mass transfer is enhanced at these scales), capacitive currents are small and they would also reduce the probability to multi-nanoparticle impacts, which could complicate the analysis of signals [237]. It is also worth to mention that the employed instrumentation plays an important role to get appropriate signals and the use of suitable electronic filters is important [238]. Since there is a growing demand for reliable, high-throughput and miniaturised technologies for single-entity studies, the nanoimpact method has been implemented in areas as diverse as electrocatalysis, materials science, bioelectrochemistry and (bio)sensing [233,239]. The nanoimpacts method is usually divided in three major categories depending on the electrochemical process underlying the discrete collisions on the electrode (Figure 8). The electrochemical response observed when individual particle collides with an electrode can be due to: (a) direct electrochemical oxidation or reduction of the particle; (b) enabled by an electrocatalytic reaction; or (c) blocking of electron transfer. This technique enables the detection of single entities such as nanoparticles, cells, vesicles or viruses. Single-molecule detection has also been demonstrated for proteins or DNA molecules [240].

### 4.1. Electrochemical Oxidation or Reduction of Colliding Particles

In the first case, the electrode is maintained at a potential suitable to drive the oxidation or reduction of the colliding particle, and transient currents would arise from the stochastics impacts typically with a spike-like chronoamperogram [241]. This technique is usually called anodic (for oxidation) or cathodic (for reduction) particle coulometry. The amount of charge transferred during each single collision event could provide important information on the particle properties such as size, surface charge and lifetime. The frequency of collisions is usually dependent on the particle concentration in the solution [242]. The direct electrochemical detection of nanoparticle impacts opened a window of opportunity for biosensing applications through the study of collision events of labelled nanoparticles [243]. For instance, Compton et al. developed a method for the detection and quantification of individual silver nanoparticles by anodic particle coulometry [241], which was later employed for the electrochemical detection of *E. coli* bacteria [244] and of influenza virus [245] at the single-entity level. In both works, single bacterium or virus were tagged with silver nanoparticles and the anodic striping charge resulting from the collision of individual entities with a carbon electrode were registered. The average spike charge determinate the extent of decoration with the nanoparticles and the frequency of impacts was used to establish a linear relationship with the nanoparticle-decorated single entities present, leading to a quantitative detection strategy.

Even though metallic-based nanoparticles are the most reported for nanoimpact electrochemistry, the random collision of other individual (bio)particles, called “soft particles”, have also been subject of study [239]. Liposomes are very useful for food and pharmaceutical industries due to their ability to encapsulate a diverse range of molecules. They have been studied by the nanoimpact electrochemical method [246]. Recent works detailed the detection of those vesicles by measuring the oxidation of vitamin C [247] and catecholamine [248] encapsulated on liposomes. The transient anodic currents were measured after the rupture of the individual vesicles and release of its redox content. Toh et al. described the direct electrochemical detection of cetyltrimethylammonium bromide-based micelles through the oxidation of the bromide content when single micelles collided with a carbon microdisc electrode surface [246]. The electrochemical detection of unlabeled single cancer cell collisions onto a gold microelectrode, only in presence of a specific surfactant was also described. The differences in cancer metabolism in comparison with healthy ones was used for the development of a direct electrochemical strategy where redox-active molecules were detected and allowed to differentiate the cells [249]. The monitoring of a single enzyme turnover due to the measurement of electrocatalytic currents resulting from a direct electron transfer between a gold ultramicroelectrode and colliding single laccase enzymes was also described [250]. The obtained results provided fundamental information about enzymatic mechanisms and enable further application to other redox enzymes. The individual detection of red blood cells under physiological conditions and the correlation with its concentration (frequency of collision) was reported [251]. The operating principle was the reduction of oxygen coming from the cells suffering hemolysis during the impacts. The transient signals were enhanced in the presence of hydrogen peroxide.

### 4.2. Electrocatalytic Nanoparticle Impacts

The electrocatalysis of a redox molecule by a nanoparticle when it collides with a non-catalytic electrode surface have also been described [252,253,254]. In this case, a catalytic reaction only occurs when the nanoparticle is in contact with the electrode surface leading to an electrocatalytic amplification (ECA). The stochastic collision events occurring at the electrode result into a step-like or spike-like chronoamperogram depending if the particles is adsorbed onto the electrode after the collision or if it can move back to the bulk solution [3,242]. Typical redox indicator species are used for the ECA strategy such as hydrazine [255,256], sodium borohydride [257], hydrogen [258], water [254] or oxygen [259]. Regarding biosensing with bio-decorated nanoparticles, ECA strategy presents a main drawback: the adsorption of the biological species onto the nanoparticles surface usually contributes to the blocking of its catalytic surface sites required for the redox process [260]. Nonetheless, recently published works propose alternatives to overcome said limitations. Castañeda et al. reported turn-off/turn-on strategies where the specific enzymatic digestion of ssDNA [261] and microRNA [262] onto Pt nanoparticles was used to address the adsorption issue. This strategy allows to recover the active surface sites on the nanoparticle after the enzymatic digestion of the biomolecules covering the nanoparticle surface.

### 4.3. Surface Blocking by Adsorptive Nanoparticle Impacts

Stochastic electrochemical detection was also extended to the detection of discrete collisions of un-supported single biological species via a blocking effect [263]. For that purpose, the steady-state current registered at an electrode, in which a redox mediator is being continuously oxidised, suffers a staircase-shaped decrease as a consequence of single collisions onto the electrode [239]. Upon the adsorption of the colliding particle on the electrode surface, a blockage of the electrode active sites takes place. From the height and frequency of the current steps, the size and concentration of the (bio)analyte can be determined. Dick et al. proposed a method to assay single virus collisions. After individual collisions, single virus molecules are irreversibly absorbed on a Pt electrode, blocking the flux of ferricyanide to the electrode surface [264]. This strategy is not highly selective since any species in the solution that adsorbs on the electrode surface will lead to a current response. A second work from the same authors proposed an improved strategy where the signal was enzymatically amplified through the detection of the specific collision of a single virus on a Pt electrode with the aid of a glucose oxidase (GOx)-functionalised specific antibody [265]. Similar approaches were employed using the blocking of the electrode surface to detect other electrochemically inactive single (bio)entities such as bacteria [266,267], proteins and DNA [240].

## 5. Nanoplasmonics

Nanoplasmonics [268] is a scientific field that study optical phenomena in metallic structures at the nanoscale. In such systems, the electromagnetic radiation can be concentrated by objects that are significantly smaller than its wavelength due to surface plasmons, which are coherent electron oscillations from the metal confined to a metal-dielectric interface in response to incoming light. Plasmonic properties of metallic nanostructures are strongly dependent on characteristics such as size or geometry and very sensitive to the surrounding medium (dielectric) where they propagate. Some consequences of the nanoplasmonic effect are large electromagnetic field enhancement or a high photothermal conversion efficiency that can be exploited for several applications. It is well known that the stronger enhancements occur in specific positions of the nanostructure called hot spots. These hot spots could include inter-particle gaps in particle aggregates or edges and curvatures within a single nanoparticle among many other configurations. The ultrasmall scale where the surface plasmons are localised in nanostructures enable the interaction with small entities even achieving interactions at the single-entity level. Since the structure (material, size, shape) of plasmonic nanoobjects controls their properties, numerous fabrication methodologies [269,270] have been reported for tailored engineering of high-efficient nanoplasmonic structures such as particle aggregates, nanogap antennas [271], nanotriangles [272] among others. There are several approaches to detect the localised nanoplasmonic effect on molecules in the vicinity of the nanostructures [273,274]. This review focuses on three of the main approaches: (a) shift of the localised surface plasmon resonance (LSPR), (b) plasmon-enhanced fluorescence and (c) plasmon-enhanced scattering as in Surface-enhanced Raman scattering (SERS). All these techniques demonstrate an excellent sensitivity, being able to detect single-entity interaction events. These methodologies exploiting the plasmon enhancement enable the detection or imaging of a wide range of single (bio)entities such as molecules (nucleic acid, proteins), cells or viruses, composing a great analytical resource to study many biochemical processes. Plasmonic techniques have their own challenges and limitations, which have been described elsewhere [275]. Briefly, one of the main challenges is to place the individual entity at the hot spot where the plasmonic enhancement is maximum, which would enable the single-entity detection. This can be especially problematic with low concentrated samples, since the binding of the analyte with the hot spot could take a considerable amount of time. In some approaches, such as the LSPR shift monitoring, non-specific adsorption could also induce a LSPR shift and selectivity would be compromised as the obtained signal could be interpreted as a specific binding. Functionalisation with specific receptors could enhance the selectivity allowing the binding of only the target analyte. However, covering the nanostructure surface with a biolayer would increase the distance between the surface where the plasmon is generated and the analyte, which could result in lower sensitivity. Raman techniques address the selectivity issues since it can provide molecular recognition. For plasmin-enhanced fluorescence, labelling with a fluorophore is usually needed to achieve a high sensitivity, and that may not be practical, especially for small molecules.

### 5.1. Single-Entity Detection by Monitoring the Plasmon Band

Localised surface plasmons oscillate at a typical frequency due to the resonant excitation of free electrons in the metallic nanostructures. This effect leads to a spectral extinction peak at that frequency, which is called the plasmon band. Ultraviolet-visible (UV/VIS) spectroscopy can be used to measure the surface plasmon frequency since a typical band appears in the spectrum when the incident radiation coincides with the frequency of electron oscillations, normally in a range between 400 and 1000 nm and dependent on the material properties. Surface plasmons are affected by the refractive index of the nanostructure surroundings and, therefore, interactions such as molecular bindings on the surface could lead to small changes in the local refractive index and detectable changes in surface plasmon properties. A possible effect of this interaction is a frequency shift of the LSPR band [275]. This detection approach is label-free since the detectable signal comes from the plasmonic nanostructure. Discriminating specific from non-specific binding in complex biological media is still challenging, since the non-specific interaction could dominate the stochastic response. To minimise this issue, time-resolved studies could help to identify strong (specific) binding or to generate a kinetic fingerprint for identification of the stochastic events.

#### 5.1.1. Detection of Nucleic Acids and Proteins

**Figure 9:**
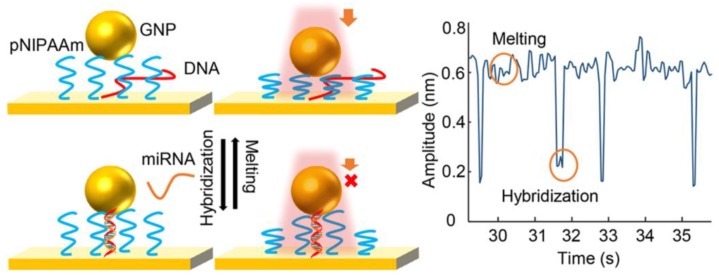
Schematic showing the high-amplitude light-induced oscillation of gold nanoparticles in absence of target miRNA (**top**) and decrease of the oscillation amplitude after formation of a DNA-miRNA rigid duplex structure in the gap between the nanoparticle and the surface (**bottom**). Monitoring the oscillation amplitude provides information about individual binding and melting events of single miRNA molecules (**right**). Reprinted with permission from [278] Copyright 2018 American Chemical Society.

The LSPR band shift approach has shown promising results for the sensitive detection of different biomolecules even at the single-entity level. For instance, different strategies have been reported for sensing of nucleic acids. The detection of single binding events of DNA to functionalised gold nanoparticles was achieved using DNA target and probe molecules attached to gold nanoparticles of 20 and 100 nm, respectively. After the biochemical binding, both nanoparticles become closer leading to a shift of the LSPR band [276]. Detection of single hybridisation events of microRNA was achieved using Au@Ag core-shell nanocubes modified with tetrahedron-structured DNA molecules. A change of 0.4 nm on the LSPR band was observed for a single hybridisation event. The system was also employed as a DNA-based logic operator showing interesting potential as biomemory by combining LSPR spectroscopy and colour imaging [277]. Recently, a smart device formed by gold nanoparticles and mechanoresponsive polymer chains working as nanooscillators attached to a gold surface was also employed for detection of single-molecules of microRNA (Figure 9). In this system, in an initial OFF state, the responsive polymers are actuated by light excitation leading to oscillation of the gap between the gold nanoparticles and the gold surface. When the hybridisation between DNA and microRNA strands occurred at the gap, the oscillation amplitude decreased, separating the nanoparticle and the surface due to the formation of a rigid dual-strand structure. Thus, the LSPR band shifted accordingly with the distance induced by the coupling effect between plasmonic nanoparticles and the gold surface [278].

Single-molecule detection of proteins was also achieved by monitoring the LSPR shift in different nanoplasmonic systems when single binding events between a protein and a receptor occur. This is normally achieved by visualising individual nanoparticles, typically by dark-field microscopy. Several systems using functionalised plasmonic silver nanoparticles [279], gold nanorods [280,281] or gold bipyramids have been reported with excellent sensitivity to detect single bindings of proteins such as streptavidin or antibodies. Combination of the typical ELISA biochemical assay with detection of LSPR shifts was also reported [282]. This system employed isolated gold nanoparticles modified with Horseradish peroxidase (HRP) enzyme molecules able to catalyse a reaction leading to an insoluble product that precipitates locally on the nanoparticle surface. This process causes a dramatic shift in the LSPR band, achieving the detection by a simple colorimetric technique of only one or a few HRP molecules per nanoparticle. Photothermal microscopy was also demonstrated to detect single binding event of a streptavidin complex on individual plasmonic gold nanorods functionalised with biotin. The LSPR shift caused by a single affinity binding on a nanorod leads to a change of the detected photothermal signal by a local temperature change. This technique is capable to detect nanoparticles as small as 1.4 nm [283].

#### 5.1.2. Cell Analysis

Monitoring the LSPR band within nanoplasmonic structures has also been used for sensing and imaging of individual cells. LSPR band can be used directly as a signal for nanoparticle tracking inside living cells or the shift of LSPR could also provide information of subcellular processes by interaction of the plasmonic nanoparticles with specific chemical species. Dark-field microscopy is normally employed as it provides single-nanoparticle sensitivity. No labelling with fluorophores is needed compared to fluorescence microscopy, which is perhaps the most employed technique for cellular imaging. Processes as diverse as the transport of single silver nanoparticles in zebrafish embryos [284], formation of lipid bilayers during exo- or endocytosis events [285], imaging and characterisation of single receptor molecules [286], transport kinetics in membrane [287] have been studied by LSPR monitoring of plasmonic nanoparticles. Intracellular p53 protein dynamics in HeLa cells was studied using a nanoscale surface plasmon-polariton fibre-tip-probe [288]. This system combines an optical fibre with a nanometre tip modified with a single gold nanorod at the tip. When the nanorod is functionalised with antibodies it can be used to sense specific targets proteins, which causes a shift of the LSPR band after the affinity binding. The tip can be inserted into single cells for localised intracellular studies. Interaction of two individual gold nanoparticles was another approach used for imaging cells. The gold nanoparticles were modified with complementary receptor-target species and when they interacted inside living cells a plasmon coupling effect was observed leading to LSPR shift. This system enabled to monitor nanoparticle tracking experiments at sub-diffraction limit distances in live cells [289].

### 5.2. Single-Entity Detection by Plasmon-Enhanced Fluorescence

Fluorescence is by itself a very sensitive technique employed in a wide number of sensing and imaging applications with capability to detect single molecules [290]. Perhaps most single-molecule sensors exploit single-molecule fluorescence in applications such as DNA sequencing [291] or immunoassays based on single enzymes [292]. Fluorescence can also be enhanced by plasmonic effects, which is usually called metal-enhanced fluorescence or plasmon-enhanced fluorescence. This enhancement can enable the use of different fluorescent labels (normally less efficient) or lead to increased detection sensitivity with interesting implications for bioanalytical applications [269]. The fluorescence enhancement can be produced by two main factors after interaction of the plasmonic structure and a fluorophore due to the confinement of the electromagnetic field and increased field intensity: (a) increment of the excitation/absorption rate, and (b) increment of the fluorophore quantum yield.

In recent years, several reports have demonstrated the use of fluorescence detection enhanced by nanoplasmonics for detection of single biological entities, especially for single-cell analysis but also with promising studies of single-molecule detection. Plasmonic nanoantenna show very strong plasmonic enhancements leading to very sensitive detection and enable the probing of only a small solution volume close to the nanoantenna. Nanoantenna are usually formed by two plasmonic nanostructures placed very close leaving a small nanogap in the middle. For instance, the use of a gold nanoantenna placed inside a nanoaperture, which authors called antenna-in-a-box, was reported [293]. This system allowed the optical detection of isolated solution volumes as small as 58 zl, with a 1100-fold fluorescence enhancement. The excellent properties of this system were exploited for detection of several biomolecules such as DNA or proteins at the single-molecule level. Formation of nanoantenna can be performed exploiting the structural versatility of DNA origami to precisely space two gold nanoparticles [271]. This system enables the detection of molecules placed in the gap (hot spot), with expected enhancements up to 5000-fold, enabling the detection of single-emitters. Direct visualisation of binding/unbinding events of DNA strands and conformational dynamics of a DNA junction was also possible with a similar nanoplasmonic system [294].

**Figure 10:**
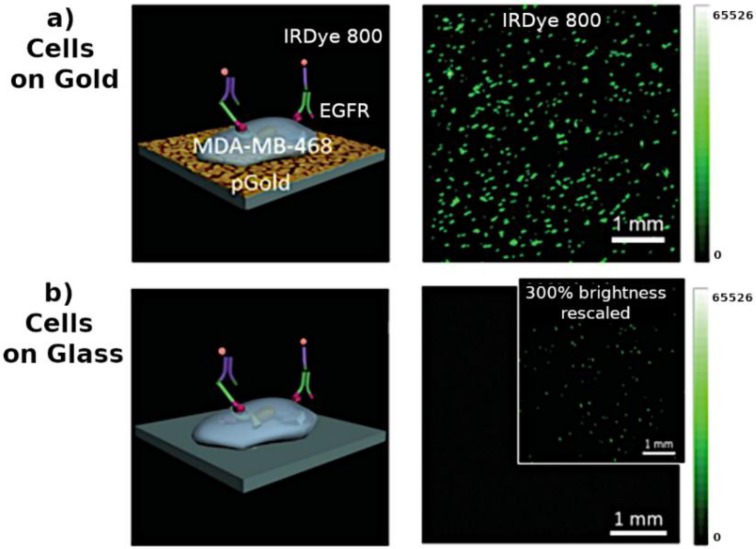
Nanoplasmonic gold platform that shows the enhanced fluorescence due to the plasmon effect in comparison to a glass surface for the detection of cellular components. Adapted with permission from [297] Copyright 2015 John Wiley and Sons.

Enhanced imaging of individual cells using nanoplasmonic fluorescence has been reported with promising results for several applications. A fluorescent metal nanoshell composed of silica spheres with encapsulated fluorophores as core and a silver thin layer as shell was employed for detection of intracellular microRNA in lung cancer cells. Single-strand oligonucleotides were attached to the metal nanoshell able to hybridise to the target microRNA molecules inside the cells, enabling the possibility of analysing microRNA distribution patterns at the single cell level [295]. Imaging of secreted proteins from stimulated single cells was also possible using a nanoplasmonic silver/silica nanostructure leading to 200-fold fluorescent enhancement [296]. A versatile system was reported using a nanostructured plasmonic gold chip (Figure 10) for multiplexed single-cell analysis of different cellular biomarkers with several fluorophores covering a wide range of emission between the visible and near-infrared frequencies [297].

### 5.3. Single-Entity Detection by Surface-Enhanced Raman Scattering

Raman spectroscopy is a very powerful scattering technique since it can provide information about molecular vibrations and therefore the spectrum recorded is a fingerprint of the molecule. Typical Raman spectra are composed of narrow peaks that also enables the identification of different analytes in complex sample mixtures. However, one intrinsic feature of Raman spectroscopy is its low sensitivity due to the low number of scattered photons with different wavelength than the incident radiation. Scattered photons from molecular vibrations can also interact with surface plasmons in metallic nanostructures, leading to a significant signal enhancement, which is called the Surface-enhanced Raman scattering (SERS) effect. The SERS effect can be roughly explained by two possible events: (a) electromagnetic enhancement (EM), based on the plasmonic properties of the nanostructured surface and (b) chemical enhancement (CE), which is based on changes in the molecular polarisability by strong adsorption of the molecule on the metallic surface. SERS enhancements are especially remarkable in metallic nanostructures formed by silver, gold or copper, although other materials have also been reported. SERS-active surfaces can be formed by colloidal nanoparticles which are usually dispersed in a solution or by solid-state substrates with a nanostructured surface [298]. It is intrinsically a label-free technique since the spectrum can be recorded directly, but molecules with strong SERS enhancements can also be used for labelling purposes. The special features of the SERS technique enable the detection of ultralow concentrations of analytes and single-molecule detection has been demonstrated in recent years. The SERS enhancement is especially strong at the hot spots and since the surface plasmons cannot travel long distances, the target molecule must be close to these structural features. Nanofabrication methodologies have been developed to create nanostructures with high density of hot spots [299], and even electrochemical roughening has been useful for in situ generation of hot spots [300] or to tune the adsorption of specific molecules by controlling the surface charge of the metallic substrate (working as electrode) [301,302].

#### 5.3.1. Cell Analysis

Cell analysis at the single-entity level has been demonstrated with SERS detection and nanoplasmonic systems. For instance, a double nanostructured system was employed for isolation and detection of breast cancer cells. Au@Fe_3_O_4_ nanoparticles functionalised with a specific aptamer were employed to capture cells through interaction between the aptamer and overexpressed proteins on the surface of cancer cells. For the detection, gold nanoparticles with SERS properties functionalised with another specific aptamer and a SERS label were employed with capability of single cell analysis [303]. Au@Au core-shell nanoparticles with a narrow nanogap between layers to create hot spots and functionalised with hyaluronic acid as receptor were employed for identification and imaging of proteins overexpressed on the surface of cancer cells [304].

Identification and discrimination of pathogenic bacteria at single-cell level based on their SERS fingerprint was also possible using silver nanoparticles prepared in situ in presence of the bacteria, with better results than using a colloidal suspension [305,306]. A nanoplasmonic system generated by assembly of silver nanocrystals in silver nanospheres was also reported for differentiation of pathogenic bacteria and with a limit of detection as low as 10 bacteria per mL in the solution [307]. Another interesting application of SERS is the possibility to detect and count between live and dead bacteria in both liquid and glass surfaces at the single cell level. This methodology proves useful in applications such as studies of antibiotic resistance of pathogenic bacteria [308]. Coupling SERS detection in microfluidic chips is also possible for online high-throughput detection of bacteria. Aggregates of silver nanoparticles functionalised with specific antibodies were employed to produce strong hot spots and capture capabilities, enabling the detection of single bacterium [309].

#### 5.3.2. Cell Imaging

SERS is also a great technique for imaging single cells and several reviews have described this topic [310,311,312,313]. Raman microscopes allow for obtaining localised detection of Raman signals with good spatial resolution (currently down to submicrometer) enabling the imaging and mapping of samples. The use of SERS-active nanosystems is an advantage to fluorescence imaging since they do not suffer from photostability issues such as fluorophores. Some of the latest advances in this technique coming from publications in the last few years will be described. Simultaneous and three-dimensional imaging of organelles (nucleus and membrane) in single HeLa cells was possible using labelled and label-free SERS detection, achieving the study of the chemical nature of nuclei and precise localisation of proteins even during apoptosis [314]. High-speed confocal Raman microscopy in combination with SERS-active gold nanoparticles were employed for imaging of cells and visualisation of cell morphology variation during induced cell death [315]. SERS imaging of cancer cells, specially targeted to differentiate from healthy cells is also an interesting application. For instance, hollow gold nanoparticles functionalised with a Raman-active label and a biochemical receptor attached by click chemistry was used to bind specifically to cancer cells for SERS imaging [316]. Antibody-functionalised nanoparticles were also employed as label for SERS imaging of cancer cells, with possibilities to study cell dynamics since the cells were able to take up the nanoparticles [317]. An interesting system where functionalised nanoparticles were self-assembled to generate hot spots, enabled the selective and sensitive SERS imaging of targeted cells and photoacoustic signals for use as a contrast agent in photoacoustic microscopy [318]. SERS tags with a core-shell structure formed by gold nanoparticles and silver nanoparticle satellites to create sensitive hot spot was recently described [319]. These tags functionalised with albumin and polydopamine were applied for imaging human glioma cells by recognition of a specific receptor in these cells.

#### 5.3.3. Proteins and Nucleic Acids

**Figure 11:**
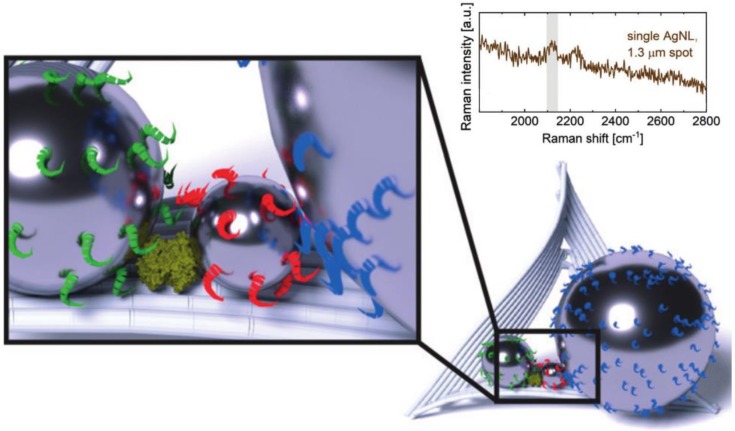
Silver nanolenses with a hot spot formed in the nanogap between two nanoparticles. Biotin receptor is able to bind one streptavidin molecule in the hot spot that can be detected by the specific surface-enhanced Raman scattering (SERS) signal. Adapted with permission from [322] Copyright 2018 John Wiley and Sons.

SERS detection of different biomolecules such as proteins and nucleic acids even achieving the single-molecule level has also been reported. Detection of a small number of p53 molecules in human serum was possible to study cancer signatures with an ultrasensitive SERS assay close to the single-molecule limit. Gold nanoparticles modified with a Raman-active label and functionalised with a receptor were used to bind different types of p53 proteins [320]. Haemoglobin was detected using isolated and immobilised silver nanoparticles. Stochastic fluctuations of the signal were attributed to the characteristic detection of single molecules, which was only possible when the molecule was situated between two silver particles, in a hot spot [321]. Silver nanolenses constructed by self-assembling of DNA-functionalised silver nanoparticles by DNA origami scaffolds were employed as a nanoplasmonic system with sensitive hot spots formed by the gap between nanoparticles (Figure 11). Single-molecules of streptavidin were placed in the hot spots and detected by SERS mapping combined with atomic force microscopy [322].

Identification of nucleobases such as adenine can be performed at the single-molecule level using colloidal silver clusters [323]. A multistep system with in situ generation of hot spots through a sandwich assay between a capture probe and a target molecule achieved the detection of DNA at sub-attomolar concentrations. Authors claim to be able to detect 10^−23^ mol, which is very close to the single-molecule level [324]. Gold-silver core-shell dumbbells were prepared and employed for single-molecule DNA detection of an anthrax oligonucleotide as a target sequence. This system was formed by two gold nanoparticles modified with different DNA probes, one of them conjugated with a Raman active dye (Cy3). In presence of a common target DNA sequence, the DNA molecules hybridised, and the gold nanoparticles formed a heterodimer. A silver shell was then growth on the gold surface with tailored thickness to generate a nanogap between the particles that enabled the single-molecule SERS detection [325]. Although possibly not at the single-molecule level, a sensitive SERS assay using gold nanoparticles was developed to study DNA methylation. In presence of methylated DNA, the nanoparticle was able to bind with a labelled-nucleoside through a single base extension reaction, causing a high SERS signal after further addition of gold nanoparticles [326].

#### 5.3.4. Tip-Enhanced Raman Scattering

Tip-enhanced Raman scattering (TERS) [327] is similar to scanning probe techniques since it uses a nanoscale tip (typically a STM or AFM tip) positioned close to the sample. However, in this case, the nanoscale tip enables the confinement of the plasmonic effect at one specific location of the sample, and scanning mode is possible for imaging applications. A Raman microscope is also employed in the setup to record the scattered light from the sample, combining the specificity of Raman spectroscopy with the high spatial resolution of scanning probe techniques. Spatial resolution about 100 times smaller than the diffraction limit of light is possible [328] since the spectra are collected only from molecules near the tip apex [329]. These special characteristics make TERS a very promising technique for single-entity imaging. For instance, single-molecule detection has been widely demonstrated, and corroborated with methodologies such as the detection of different isotopologues [330]. Coupling TERS with other techniques such as electrochemistry [331] opens the possibility to obtain even richer information about reactions and reaction intermediates with excellent sensitivity. The interested reader is referred to several reviews [328,332,333] about TERS fundamentals and applications for deeper understanding.

TERS tip are typically fabricated using metallic materials able to produce the nanoscale plasmon enhanced features. Fabrication of the tips can be performed by both top-down and bottom-up approaches [327,333,334]. It is common to use an AFM tips coated with a thin-layer of gold [335] or silver [336], which shows high plasmonic enhancement. For STM, electrochemically etched metallic tips are also employed [337]. The chemical composition, diameter and shape of the tip determine the imaging properties such as the imaging quality, enhancement factor (sensitivity) and spatial resolution. Therefore, the fabrication of highly reproducible, small size and sensitive TERS tips is a current challenge of the technique [327]. Functionalisation of TERS tips [338] can also be performed to get richer chemical information or enhance the imagng properties. For instance, the modification of a gold tip with a single or a few Nile blue molecules was possible to study electrochemical reactions at this scale by TERS [338]. It was recently demonstrated the functionalisation of TERS tips with a single CO molecule. These CO-modified tips allow for obtaining information about molecular structure and charges with atomic resolution and enhancement factors up to 10^13^ [339]. It has also enabled the study of single metalloporphyrins by imaging molecular charges, intramolecular polarisation, local photoconductivity, atomically resolved hydrogen bonds and surface electron density waves [340]. These studies demonstrate the excellent possibilities opened by the functionalisation of TERS tips.

TERS has been used for many applications in inorganic, organic and biological materials [329,333,341]. As mentioned is a technique with high potential for single-entity studies. For instance, TERS can be used to distinguish [342] and monitor submolecular conformational dynamics [343] of different porphyrin molecules that are within van der Waals contact on a metallic surface. A lateral resolution as low as 2.6 Å was achieved working at ultrahigh vacuum conditions [343]. Detection or imaging of individual proteins with TERS has allowed for studying different processes or properties such as the glycosylation status of Rnase [344], elucidation of structure and composition at the single-molecule level [345,346] enabling the identification of submolecular heterogeneities [347], or the identification of different proteins in a cancer cell membrane [348]. TERS has been used for studying the aggregation pathways of neurodegenerative peptides by detecting single amyloid fibrils at various stages of aggregation [349], which demonstrates the great applicability of the technique to study complex processes with biomedical interest. Nucleic acid imaging and detection has also been demonstrated by TERS for different applications such as probing single-strands of RNA [350] and DNA [351], identification of ssDNA and dsDNA systems that could be useful for the detection of DNA hybridisation [352]. The molecular characterisation of DNA double strand breaks, which are significant lesions that could lead to genetic defects and cell apoptosis, was also reported [353]. The technique is also promising for sequencing applications, and it has been tested for sequencing individual DNA or RNA strands as in Tobacco mosaic virus [354]. Two individual DNA bases (adenine and thymine) were unambiguously distinguished with spatial resolution down to 0.9 nm, opening the possibility to differentiate in real space, individual DNA bases in coupled base pairs [355]. TERS has also been used to probe the surface of single cells [356], subcellular entities such as large single mitochondria [357], or to detect hemozoin crystals in the digestive vacuole of a sectioned malaria-infected cell at a spatial resolution less than 20 nm [358]. Identification and discrimination of two pathogenic viruses at the single-entity level was also achieved with TERS in combination with chemometric analysis [359].

## 6. Nanomachines

Nanomachines are nanoscale objects that can perform some mechanical work (actuation, motion, propulsion) after an initial stimulation. The nomenclature of nanomachines has been quite heterogeneous in the literature with names including machines, motors, walkers, rockets, robots, or jets among many others, and sometimes they are used indistinctively to name the same type of nanomachine. This review focuses on molecular machines (biochemical and artificial), composed by molecular systems, and propelled nanomotors [360], typically formed by nanomaterials that can be transported in a medium by means of some (chemical or physical) input to generate the motion. Nanomachines is a quite new research area, but promising applications have already been demonstrated. These nanomachines provide special functions to a system, but it is still needed to have a technique able to measure the nanomachine by itself or the nanomachine output. The appropriate detection technique will depend on the type of nanomachine, dimensional scale and the function they provide (i.e., it is not the same to measure a molecular system or a micro/nanoparticle). For instance, propelled motors in the microscale range could be visible with an optical microscope, but at the nanoscale range more complex systems such as electron microscopy, dark-field optics or confocal laser microscopy [361] could be essential. High resolution fluorescence microscopy is also able to provide single-molecule imaging [362] or another tracking techniques [363] have also been reported. Molecular machines need of course high resolution techniques [364] to be visualised such as high-speed AFM [365], although in many bioanalytical applications, the actuation of these nanomachines generates amplified signals that can be measured with more conventional techniques. The potential of molecular machines and propelled nanomotors for biosensing/bioimaging with special emphasis at the single-entity level will be described.

### 6.1. Molecular Machines

Molecular machines [366,367] are composed of a number of molecular components that can output some kind of mechanical work as a result of an appropriate external stimulation (input). A chemical reaction is typically used as the input signal to start the mechanical work of the nanomachine, although the use of external inputs such as electrons [368], photons [369,370] or magnetic fields [371] could have several advantages such as avoiding the use of chemical fuels to increase biocompatibility. The output of the nanomachines could include rotation, molecular rearrangements (conformational changes) or controllable motions in interlocked molecules. They have a bright future in the nanotechnology world and it seems clear that they will form part of a number of advanced nanoscale devices to solve complex issues in the coming years. Due to the importance and potential of the topic, the Nobel Prize of Chemistry in 2016 was awarded to researchers working in this field.

Biological molecular machines are formed by biochemical components usually forming part of complex biological systems. Perhaps the most studied are biological motors that convert chemical energy to stepwise linear or rotary motion and are involved in many functions such as muscle contraction or intracellular transport. Some examples [372,373] include motor proteins of the myosin or kinesin families, adenosine triphosphate (ATP) synthase, flagella motors in bacteria, etc. Engineering biological machines has led to the generation of smarter biological systems with enhanced features respect to the natural ones [374]. Biological machines usually work only in specific and mild physiological environments since they can lose their functions in other more aggressive environments.

Artificial molecular machines [366] are synthetic machines formed by molecular systems. They were initially inspired by biological machines to mimic their functions [375], but the horizon has now further expanded with advanced synthesis methods and smart ideas. In general, they can tolerate a more diverse range of conditions than biological machines. Some examples of artificial molecular machines include nanocars [368] or molecular elevators [376] formed by a combination of molecular systems or rotaxanes, respectively. DNA walkers are a special kind of nanomachines formed by DNA molecular systems that can walk by an underneath track by effect of several steps of conformational transitions, hybridization events or enzymatic reactions. The numerous structures that can provide DNA molecules and the versatility to have short or long, single or double strands have enabled a vast number of walking actions to DNA molecules to provide different mechanical or chemical functions. They have been widely employed in bioanalytical applications [377].

### 6.2. Propelled Nanomotors

Propelled nanomotors [378] are nanoscale solid-state objects that can travel in a medium by effect of a stimulus. They were first reported with dimensions at the microscale but reducing the dimensions to the nanoscale could enhance their properties such as better biocompatibility, efficient uptake by cellular systems or a higher collection efficiency by increased surface area. Due to these reasons, numerous propelled nanomotors have been developed in recent years for different applications and with special relevance for interaction with single cells. In a similar way to other type of nanomaterials, they can be functionalised with different chemical or biochemical modifications to provide new capabilities such as cargo transporting and release or for detecting specific target entities. Propelled nanomotors can be driven in a solution by different propulsion methods making use of catalytic reactions, ultrasound, magnetic fields, electrical signals or photons. Fabrication methodologies have to be tailored considering the propulsion method since the nanomotor properties have to be generally different for different propulsion methods. The most important features of nanomotors propelled by catalytic reactions, ultrasound and magnetic fields are briefly introduced since they are typically the most employed. To get deeper understanding of fabrication and propulsion methods, several reviews have been published where these approaches and many examples are discussed [360,379,380].

#### 6.2.1. Catalytically Propelled Nanomotors

The first reported propelled micro/nanomotors were perhaps driven by catalytic reactions. These nanomotors are fabricated with a material that will be able to produce a catalytic chemical reaction to induce the movement. This action can be generated by gas bubbles formed by the reaction (if O_2_ or H_2_ are products of the reaction) or by a concentration gradient (self-phoretic effect). This type of nanomotors usually have a heterogeneous structure where the catalytic material is placed only at one end of the nanostructure to control the motion towards one direction. More unconventional structures have also been reported to increase the directional control during the movement. The most known examples are nanomotors formed with a platinum tail, which is able to catalyse the oxidation of H_2_O_2_ to produce microbubbles of O_2_ inducing the motion. Different materials and different structures, sizes or shapes have been described to provide tailored characteristics and applications and different chemical fuels can be used depending on the material properties [381]. Nanomotors functionalised with enzymes can also be propelled by means of the enzymatic reaction and generally can work in more biocompatible environments [382]. Glucose that is found in many living biological systems can fuel enzymatic nanomotors [383] supplying an endogenous fuel.

#### 6.2.2. Ultrasound Propelled Nanomotors

In many cases, the use of a chemical fuel for the propulsion is not very appropriate since it can perturb the environment where the nanomotor moves, especially using aggressive fuels such as H_2_O_2_. Alternative approaches have been described to equip nanomotors with less invasive propulsion methods such as the use of high-frequency (acoustic) waves [384]. Ultrasounds are widely employed in clinical setting since it is not harmful for living organisms and can be used in vivo applications. Nanomotors propelled by ultrasound need specific asymmetric designs that are usually solved by fabricating nanostructures with one convex and one concave ends, designs often based on nanowires. The motion is generated by the self-acoustophoresis effect, which comes from the local pressure gradient formed in the concave end pushing the nanodevice forward.

#### 6.2.3. Magnetically Propelled Nanomotors

Low magnetic fields are compatible with cells and tissues and thus they have been used as a way to control the movement of nanomotors with promising expectative for in vivo studies. The magnetic field can be used with two main purposes: as the driving force of the propulsion or for the directional control of nanomotors propelled by other means [385]. Some part of the nanomotor structure must be composed of magnetic material in order to respond to the magnetic field, being iron and nickel the most employed.

### 6.3. Single-Entity Detection with Nanomachines

Nanomachines are interesting tools for bioanalytical applications since they can provide features such as increased sensitivity (by amplification), cargo transporting, localised targeting and delivery, ability to pass (bio)membranes and biocompatibility. However, just a few studies where single-molecule detection of biological entities has been achieved using nanomachines have been reported. Both propelled nanomotors and some molecular nanomachines have been successfully applied inside individual cells. Therefore, most applications at the single-entity level are for single cells, although single-biomolecule detection has also been reported. Furthermore, most bioanalytical applications of molecular machines have been reported with DNA nanomachines such as walkers [377]. In any case, we think that the promising characteristics of these nanosystems will place them as an important tool in future biosensing and bioimaging applications when a vast number of single-entity studies exploit these characteristics.

#### 6.3.1. Single-Entity Detection with Molecular Machines

DNA nanomachines, specially walkers have been used in different biosensing system exploiting signal amplification to achieve the ultrasensitive detection of different (bio)analytes. Even though single-molecule detection is still difficult with these systems, recognition of single-base mutations or single nucleotides variants have been reported. For instance, a two-wheel drive-based DNA nanomachine formed by one signalling recognition probe, one label-free recognition probe and one driving primer was employed for sensitive detection of cancer-related gene. The nanomachine is activated by DNA hybridisation, which starts a wheel driving by polymerisation/nicking/displacement cycles. Even though single-molecule detection was not possible, this system was able to recognise single-base mutations [386]. Nuclease-based reactions have been employed to actuate DNA walkers in many applications. A stochastic walker that can move on a spherical nucleic acid three-dimensional track propelled by digestion of hybridised DNA by exonuclease III was reported for bacteria detection. The walking motion can be monitored at the single-particle resolution using TIRF microscopy. This system can provide great sensitivity exploiting the signal amplification triggered by the DNA motion [387]. A three-dimensional DNA nanomachine actuated by toehold-exchange, which is a method for fast and reversible strand displacement reactions, was able to discriminate single nucleotide variants [388]. A DNA nanowalker formed by gold nanoparticles that can move by nicking endonuclease along a 3D track was able to release payloads from substrate cleavage. The nanomachine is very promising for biosensing since it can be tailored to achieve the rapid, isothermal and homogenous signal amplification for specific nucleic acids by incorporating a toehold exchange mechanism [389]. Another DNA-based nanomachines have also been used for sensing. For instance, a self-assembled DNA-based nanosystem combining a dumbbell-shaped frame, a cylindrical core and a mobile ring was employed for molecular interrogation being able to evaluate base stacking adhesion and detection of a soluble nucleic acid viral genome mimic. The motion of the nanosystem ring can be induced by a transition between single-stranded DNA to double-stranded DNA which show different rigidity, enabling its use as a mechanism for molecular detection at the ring-frame interface [390].

Biological-based nanomachines such as the Phi29 DNA packaging motor, which combines the functions of a nanomachine actuated by ATP [391] and a nanopore have been employed for the study of single-molecules in a nanopore sensor [392,393]. Bacterial flagellar motor that works as an ion-driven rotary motor by coupling the ion flow to torque generation through the Motility protein B (MotB) was employed for studies of protein stoichiometry, dynamics and turnover of MotB with single-molecule precision [394].

**Figure 12:**
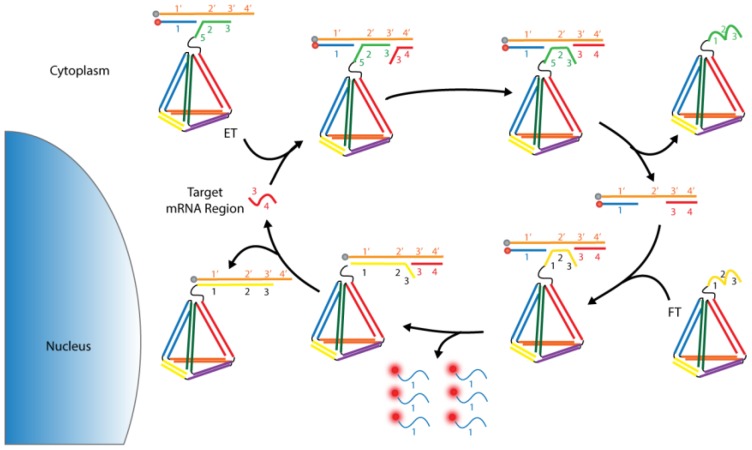
Schematic showing a 3D DNA nanomachine initiated by interaction with microRNA (analyte) leading to an amplified release of signaling molecules for intracellular detection. Adapted with permission from [396] Copyright 2018 American Chemical Society.

Molecular nanomachines are very appropriate for manipulation or sensing in single cells. For instance, the use of a molecular machine with short peptide addends able to drill an open a hole in cell membranes after activation by UV light was reported. Functionalised nanomachines can selectively target specific cell-surface recognition sites and be used for application such as delivery, release of species from cells or induce cell death (by apoptosis or necrosis) [395]. The detection of intracellular microRNA has great relevance in health studies since it can be a good biomarker of cancer initiation. Several DNA nanomachines have been used for detection of microRNA in single cells exploiting the good biocompatibility and providing signal amplification for sensitive detection. For instance, a molecularly engineered entropy-driven 3D DNA amplifier (Figure 12) initiated by interaction with specific microRNA target was used with excellent results in terms of sensitivity [396]. The autonomous walking of a DNAzyme motor system on gold nanoparticles can be started by intracellular interaction with target microRNA leading to release of numerous signalling molecules for sensitive detection of microRNA in individual cancer cells [397]. A DNA nanomachine powered by endogenous ATP molecules can be autonomously operated inside cells without auxiliary additives. The nanomachine is triggered by binding of intracellular miRNA target molecules and progressively operates on an established DNA track via intramolecular toehold-mediated strand migration and internal ATP binding, resulting in amplified release of dye-labelled substrate used for fluorescent imaging of the cells [398]. A similar system but entropy-driven was previously reported by the same authors [399].

Mapping of spatial and temporal pH changes inside living cells was also possible using DNA nanomachines triggered by protons by using a Förster resonance energy transfer (FRET) detection strategy [400] or using two nanomachines operating simultaneously in a single cell to map pH gradients in different subcellular environments [401].

#### 6.3.2. Single-Entity Detection with Propelled Nanomotors

Propelled motors at micro and nanoscales have been employed in different biosensing/imaging applications. Some general reviews on these devices in topics such as propelled affinity biosensors [402], biosensing [379] or applications in biomedicine [403,404] have been reported. Functions as delivery, capture or transport of entities can help to enhance the properties of biosensing and bioimaging fields. The movement around the sample allows for increasing the volume of sample probed in comparison to typical surface-based bioassays, which would result in more sensitive assays by enhancing the biomolecular interactions. Also, new sensing approaches can be opened due to the specific properties of propelled motors. For instance, motion-based detection [405] of biomolecules by distance travelled or changes in speed of propelled motors has been reported [406]. Although these applications were not at the single-entity level, it seems reasonable to think that advances in propelled nanomotors could enable the binding of small single-entities (molecules) which in combination with their intrinsic features (motion, cargo transport, etc.) could push forward the biosensing field in the near future. In any case, some applications of propelled micro/nanomotors at the single-entity level have been reported, specially based on interactions with individual cells. Ultrasound propelled micro/nanomotors have been demonstrated as very useful tools to provide different functions in individual living cells. For instance, they have been used for intracellular delivery of biomolecules after the evidence that efficient acoustic propulsion of rod-shaped gold nanomotors can be achieved inside HeLa cells [407]. Meanwhile, the fabrication of nanowires modified with a rolling circle amplification DNA strand to attach siRNA, propelled by ultrasound excitation for siRNA delivery to the cell was also reported. This RNA was responsible for gene-silencing the formation of specific proteins inside the cell [408]. Similar systems were employed for intracellular delivery of enzymes (caspase-3) to the cytosol of cells by releasing the cargo with a pH-responsive polymer coating the nanomotor [409] or delivery of Cas9-RNA complex released inside the cells mediated by the high concentration of glutathione in tumour cells [410]. These smart cargo transporting systems are able to detect different chemical environments inside single cells and deliver the specific cargo when the functional modification sense those environments. Different propulsion methods have also been applied for targeted cargo delivery to individual cells. Gold nanowires moved by electric field (using electrophoretic and dielectrophoretic forces) were employed for precise delivery of cytokine and activate signalling in a targeted single cell with subcellular resolution [411]. Magnetic fields applied to nickel nanowires were also demonstrated for cell manipulation, achieving the transport of individual cells and positioning one cell on top of other cell [412]. Recognition of cell membranes by the propelled nanomotors can be exploited for other functions. Near-infrared (NIR) light-powered nanomotors functionalised with a macrophage cell membrane were able to seek actively and recognise cancer cells. When the nanomotor bound to the cell membrane the photothermal effect caused by the NIR excitation perforated the cell membrane, which could have interesting applications for molecular injection [361].

**Figure 13:**
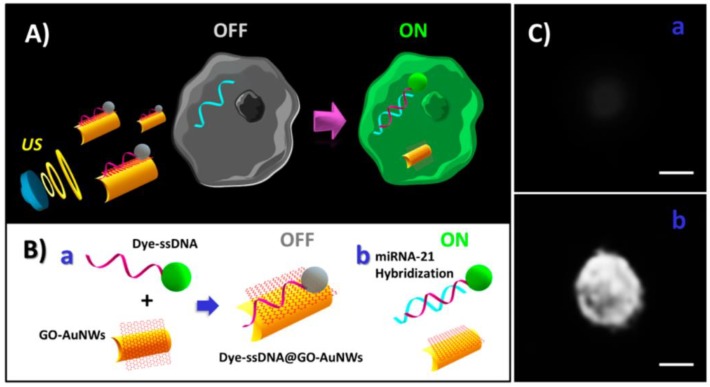
(**A**) Schematic showing the ultrasound-propelled nanomotor developed for intracellular detection of microRNA. (**B**) Schematic showing the functionalization of the gold nanowire nanomotor with the dye-labelled ssDNA/graphene oxide composite (**a**) and fluorescence recovery after miRNA hybridization (**b**). (**C**) Fluorescence images of a single cell before (**a**) and after (**b**) incubation with the nanomotor. Adapted with permission from [413] Copyright 2015 American Chemical Society.

Intracellular biosensing at the single-cell level of target microRNA in cancer cells has been demonstrated using ultrasound propelled gold nanowires capable of penetrating intact cancer cells (Figure 13). These nanowires were coated with dye-labelled ssDNA/graphene oxide composite. The close placement of the graphene oxide to the fluorescent label resulted in quenching of the emission. When the nanomotor was introduced into the cell and the nucleic acids bound to the target microRNA inside the cell, an enhancement of the fluorescent was observed due to the higher separation between the graphene oxide and the fluorescent label, avoiding the quenching [413].

SERS is a technique capable of single-molecule detection, especially when highly enhanced plasmonic scattering occurs in hot spots of nanostructures. Nanomotors with plasmonic properties have also been developed and applied to sensing of cells. For instance, a SERS nanomotor fabricated with a core-shell structure of Ag nanowires@SiO_2_ was able to move induced by excitation with UV light of the photocatalytic AgCl tail [414]. The nanomotor motion led to aggregation of nanomotors creating hot spots and enhancing the detection sensitivity of MCF-7 breast cancer cells. Although this example is probably not at single-cell level, it seems clear that the use of materials with enhanced hot spots could easily provide this level of sensitivity. Nickel nanotubes modified with plasmonic silver nanoparticles and controlled by magnetic field were also employed to target single live animal ovary cells. The nanomotors in contact with the cell membrane were able to induce the sensitive SERS signal of characteristic lipids and proteins coming from the cell [415].

## 7. Summary and Outlook

The nanoscale sensing and imaging approaches described in this review show promising results for single-(bio)entity studies. The detection of different single-(bio)entities has been reported with special emphasis on individual cells but also reaching the single-molecule level. These approaches allow the development of sensing devices with the ultimate sensitivity and the possibility to visualise rare events within a sample. Thus, addressing analytical problems from a single-entity viewpoint provides richer information and a more complete picture of the studied system. Nanoprobes allow localised measurements with very high spatial resolution and non-invasive sensing for in vivo studies. They can provide multifunctional information of the individual entities such as morphology, surface charge or electrochemical activity and by mapping the surface is even possible to visualise sub-entity heterogeneities. However, the extension of the technique for the analysis of small single (bio)molecules (e.g., proteins or nucleic acids) and specially in complex biological media remains challenging. Solving the inherent issues of working with these complex samples would help to extend these approaches to study more delicate biological mechanisms. Nanopores have reached an interesting stage of development and demonstrated a great capacity for detection of single entities, with commercial devices using this technology released to the market for sequencing applications. Nonetheless, some challenges still remain in nanopore sensing: low selectivity of conventional nanopores, fast translocation speeds of small molecules and low throughput for samples at low concentrations. Some advances to address these issues have been reported in recent years. Development of novel nanofabrication methods to achieve the precise and tuneable functionalisation of nanopores could make an important impact and open up the nanopore sensing to new applications. Nanoimpacts have shown promising results for studies of collision of single particles, and the bioanalysis applications are practically at the beginning. New nanoimpact methods for increasing the biochemical selectivity (specially in complex physiological media) or the expected widespread use of nanoscale electrodes in the next few years in combination with advances in instrumentation (to discriminate smaller faradaic currents) will be important for enhancing the applications of this approach to detect and quantify a vast number of single (bio)entities. Plasmon-enhanced optical techniques by nanomaterials provide a great sensitivity and have already demonstrated good results in the detection of single-entities, even reaching the single-molecule level. Next developments will have to search for ideal nanoplasmonic structures with very sensitive hot spots that enable single-molecule detection. It can be expected that novel nanofabrication techniques could allow the precise synthesis of complex metallic nanostructures with special plasmonic features. New efficient strategies to improve the positioning of the entity close to the hot spots would also be needed, and the use of appropriate receptors could provide specificity to the detection. Nanomachines have shown interesting functions at the nanoscale. However, no many systems have been used for bioanalytical applications such as sensing and imaging at the single-entity level. Mainly, DNA nanowalkers that can perform some mechanical motions for amplified release of signalling molecules are the most known. However, molecular machines can perform different kinds of nanoscale mechanical functions, and in many cases, they are triggered by interaction with single molecules. Therefore, the development of sensing applications employing nanomachines at molecular scale could play an important role in the future of the biomedical field if these systems can be precisely controlled.

Despite all of the challenges for single-entity detection, different powerful nanoscale tools are being developed and successfully applied for (bio)sensing and imaging at the single-entity level, placing the analytical chemistry field in the frontier of the knowledge.
